# Self-enhanced multicolor electrochemiluminescence by competitive electron-transfer processes†

**DOI:** 10.1039/d0sc00853b

**Published:** 2020-04-17

**Authors:** Silvia Voci, Romain Duwald, Stéphane Grass, David J. Hayne, Laurent Bouffier, Paul S. Francis, Jérôme Lacour, Neso Sojic

**Affiliations:** University of Bordeaux, Bordeaux INP, ISM, UMR CNRS 5255 33607 Pessac France neso.sojic@enscbp.fr; University of Geneva, Department of Organic Chemistry Quai Ernest Ansermet 30 1211 Geneva 4 Switzerland; Deakin University, School of Life and Environmental Sciences, Faculty of Science, Engineering and Built Environment Waurn Ponds Victoria 3216 Australia

## Abstract

Controlling electrochemiluminescence (ECL) color(s) is crucial for many applications ranging from multiplexed bioassays to ECL microscopy. This can only be achieved through the fundamental understanding of high-energy electron-transfer processes in complex and competitive reaction schemes. Recently, this field has generated huge interest, but the effective implementation of multicolor ECL is constrained by the limited number of ECL-active organometallic dyes. Herein, the first self-enhanced organic ECL dye, a chiral red-emitting cationic diaza [4]helicene connected to a dimethylamino moiety by a short linker, is reported. This molecular system integrates bifunctional ECL features (*i.e.* luminophore and coreactant) and each function may be operated either separately or simultaneously. This unique level of control is enabled by integrating but decoupling both molecular functions in a single molecule. Through this dual molecular reactivity, concomitant multicolor ECL emission from red to blue with tunable intensity is readily obtained in aqueous media. This is done through competitive electron-transfer processes between the helicene and a ruthenium or iridium dye. The reported approach provides a general methodology to extend to other coreactant/luminophore systems, opening enticing perspectives for spectrally distinct detection of several analytes, and original analytical and imaging strategies.

## Abstract 2

Controlling electrochemiluminescence (ECL) color(s) is crucial for many applications ranging from multiplexed bioassays to ECL microscopy.

## Graphical abstract

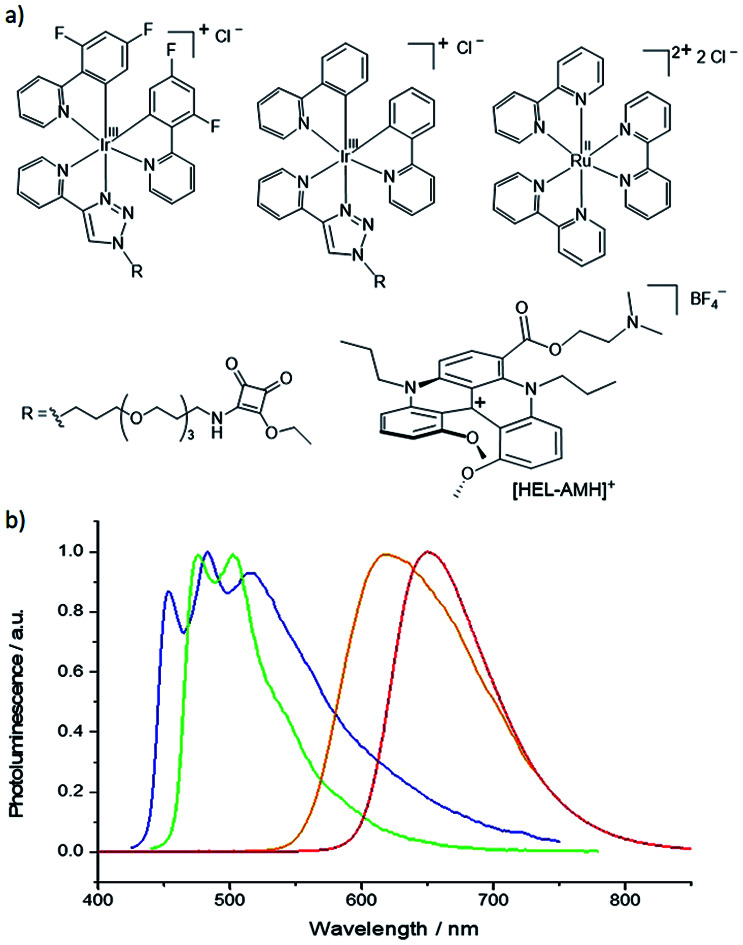

## Introduction

Electrochemiluminescence (ECL) is the light emission resulting from an initial electron-transfer reaction occurring in the vicinity of the electrode.^1,2^ The discovery of ECL emission in aqueous solution with an efficient ‘coreactant’ reagent has led to successfully commercialized bioassays for the detection of clinical biomarkers, biological warfare agents and foodborne pathogens.^3–5^ The coreactant is a sacrificial molecule that is added to the solution containing the luminophore. In the classic ECL mechanistic pathway, both molecules, the luminophore and the coreactant, have specific and separate functions. The coreactant that is either oxidized or reduced is irreversibly consumed due to a bond-breaking reaction whereas the luminophore is typically regenerated during the ECL process.^4,6^ The function of the coreactant is to provide sufficiently energetic radicals to undergo exergonic intermolecular electron-transfer with the luminophore in order to reach the electronically-excited state.^7–10^ Tri-*n*-propylamine (TPrA) and several closely related tertiary amines are the most efficient coreactants to enhance ECL intensity in aqueous solution.^9,10^ Thus, electron-transfer is at the core of the ECL phenomenon. A thorough understanding of the underlying principles of the associated inter- and intra-molecular electron-transfer reactions and reactivity is necessary to propose novel ECL strategies.

Since light is the final output of ECL, original concepts and applications have been developed exploiting the different properties of light, such as surface plasmon resonance ECL,^11^ circularly-polarized ECL,^12^ light-emitting devices,^13,14^ photoinduced ECL,^15^ and ECL interferometry.^16^ Recently, ECL has been used as a microscopy technique to image latent fingerprints,^17^ single micro- and nano-particles,^18–22^ cells,^23,24^ cellular membranes,^25^ and membrane proteins.^26,27^ Most of these applications rely on the use of ruthenium(ii) polypyridyl complexes as luminophores, especially ruthenium(ii) tris(bipyridine), and more recently on *ortho*-metalated or heteroleptic cationic iridium complexes.^28–35^ Indeed, the emission of such iridium(iii) complexes is tunable at more hypsochromic wavelengths compared to the typical orange/red color resulting from the ruthenium complexes.^31–34,36^ The ability to tune the emission color(s) has recently generated wide interest due to potential applications ranging from multiplexed bioassays to ECL microscopy.^28–31,37–42^ However, the effective implementation of such strategies is constrained by the limited number of luminophores that combine water solubility and a high ECL efficiency in aqueous media. In this context, multicolor ECL emission has been reported using a combination of different organometallic ruthenium(ii) and iridium(iii) luminophores, mainly in organic media.^28–30,41^ The concomitant ECL emissions of several luminophores have been described by controlling the energetics of the luminophore.^30,43,44^ Ding and coworkers have explored a different approach with a specifically designed iridium complex emitting at three wavelengths in acetonitrile.^45^ Nanoparticles have also been used for this purpose.^39^ For example, a hybrid system consisting of PbS nanocrystals and a BODIPY capping ligand was proposed to generate highly efficient dual emissions.^46^ However, organic molecules offer a promising alternative due to their structural versatility that allows to tune precisely the optical and electronic properties. A large variety of such organic molecules have been designed and prepared to investigate their ECL properties, almost exclusively in organic solvents.^47–54^

**Fig. 1:**
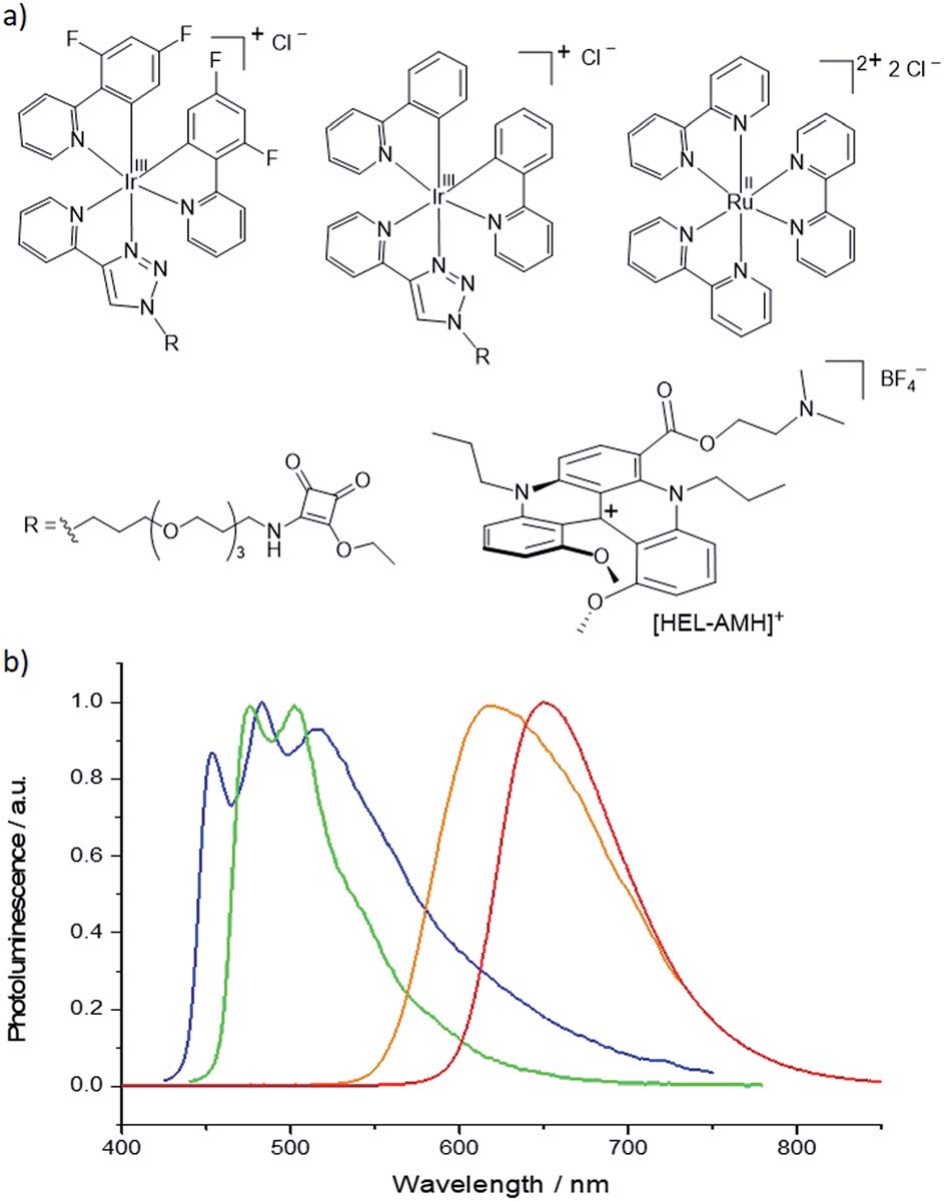
(a) Molecular structures and (b) photoluminescence emission spectra of the ECL luminophores. From left to right: [Ir(df-ppy)_2_(pt-R)]^+^ (blue curve/emitter), [Ir(ppy)_2_(pt-R)]^+^ (green curve/emitter), [Ru(bpy)_3_]^2+^ (orange curve/emitter) and the dual helicene noted **[HEL-AMH]+** (red curve/emitter). R = TOxT-Sq (a trioxatridecane chain with squarate amide ethyl ester).

In this work, we demonstrate that both functions (*i.e.* the coreactant, which generates energetic radicals and the luminophore, which is thus “electro-excited” and emits light) can be decoupled and accomplished sequentially in a single molecule in order to control multicolor ECL emissions in water. Herein, we report the first self-enhanced organic ECL dye that integrates successfully both coreactant and luminophore functions. The molecular scaffold is based on a cationic diaza [4]helicene (Fig. 1a). With the goal of obtaining multicolor ECL emission, we selected the archetypical [Ru(bpy)_3_]^2+^ complex and also two iridium(iii) luminophores that emit at shorter wavelengths (Fig. 1b) to avoid Förster resonance energy transfer (FRET). Hence, we investigate competitive highly exergonic intra- and inter-molecular electron-transfer reactions between different luminophores. Finally, we demonstrate that concomitant multicolor ECL emission ranging from red to blue with tunable intensity can be obtained by intermolecular electron-transfer processes between the electron-donor helicene radicals and all three electron-acceptor organometallic complexes.

## Results and discussion

**Scheme 1:**
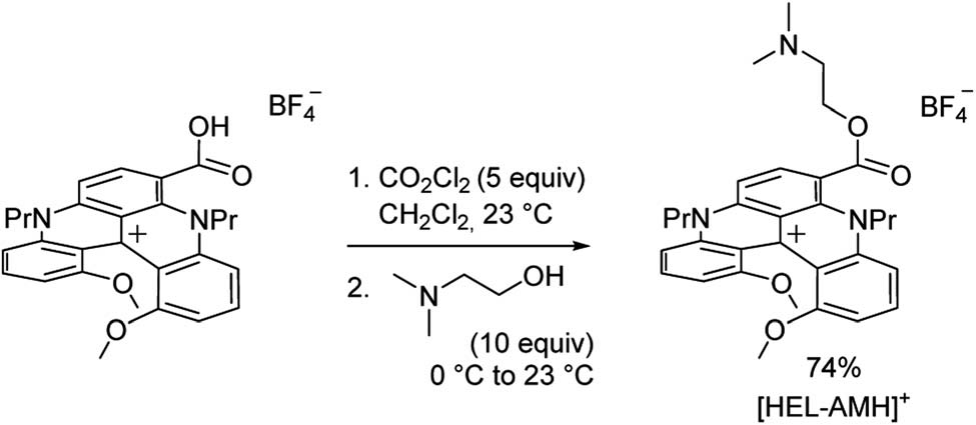
Synthesis of water soluble diaza [4]helicene **[HEL-AMH]+**.

Cationic helicenes are *ortho*-fused polyaromatics that combine the main properties of triarylcarbenium ions with the structural features of (chiral) helicenes.^55,56^ These dyes and luminophores are readily prepared,^56^ and can be further functionalized *via* versatile late stage synthetic strategies that allow the regioselective introduction of various functional groups at the periphery of the helical core.^57–61^ We selected this family of luminophores because it allows fine-tuning not only the photophysical and redox properties of the luminophores but also their solubility in aqueous media.^56,62^ More specifically, a diaza [4]helicene bearing a tertiary dimethylamino-terminated group, denoted **[HEL-AMH]+**, was designed and prepared. In this dual-function molecule, HEL and AMH refer to the [4]diaza core and to the dimethylamino moiety, respectively. It was foreseen that the core helical structure would (i) remain a red-emitter distinct from the emission of model ruthenium and iridium complexes,^57^ (ii) integrate easily the coreactant function as a substituent,^58,60^ and (iii) exhibit reasonable solubility in aqueous media. In terms of synthesis, the central helical core was functionalized at position 6 with an ester group featuring a dimethylamino moiety as its terminal end. Previously, the synthesis of the diaza [4]helicene carboxylic acid was reported (Scheme 1).^63^ Herein, this compound was converted to the corresponding acyl chloride with CO_2_Cl_2_ and subjected to a nucleophilic substitution reaction at 0 °C by slow addition of an excess of *N*,*N*-dimethylethanol. The corresponding ester **[HEL-AMH]+** was isolated after purification by flash column chromatography in 74% yield.

**Fig. 2:**
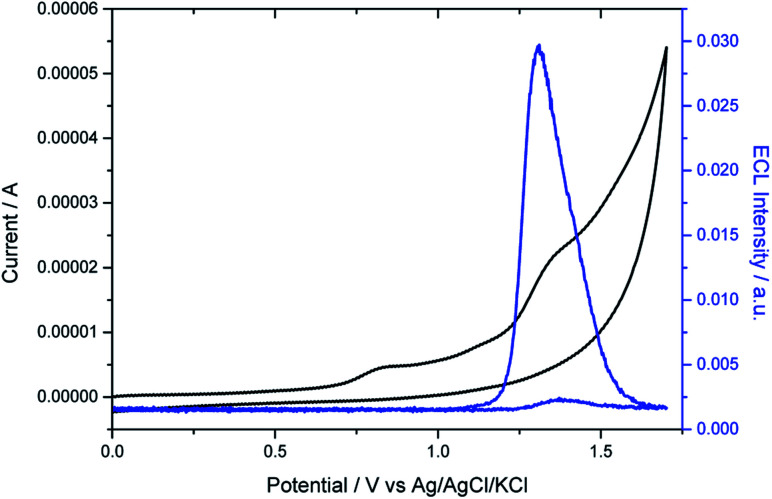
Voltammetric (black curve) and ECL (blue curve) signals of 10^−4^ M **[HEL-AMH]+** in PBS on a glassy carbon (GC) electrode. Scan rate: 0.1 V s^−1^.

As expected, the dimethylamino group improved drastically the aqueous solubility of the luminophore (Fig. S9 and S10†) and its role as coreactant was tested. The electrochemical characterization of the helicene shows two irreversible oxidation waves in PBS (Fig. 2). The first wave occurs at 0.8 V, which can be attributed to the oxidation of the dimethylamino AMH moiety (reaction (1)). Indeed, the irreversible oxidation of TPrA has been reported previously at a similar potential in aqueous media and we can assume a mechanism analogous to the one described for TPrA.^64^ The oxidation of the deprotonated dimethylamino group generates the dication radical [HEL-AMH˙]^2+^ (reaction (1)). The latter deprotonates rapidly to form locally the monocation radical [HEL-AM˙]^+^, which is a strong reductant (reaction (2)). The second irreversible wave peaking at 1.33 V (Table S1†) corresponds to the oxidation of the diaza core. To confirm this assignment, another water soluble diaza [4]helicene [HEL-SO_3_H]^+^ was also prepared (79% from the carboxylic acid precursor, see ESI†). In that case, the sulfonic group is electro-inactive in the investigated potential window and only the oxidation of the diaza core is visible at 1.3 V (Fig. S13†). Through a simple comparison of the voltammetric behavior of both [4]diaza complexes, we can therefore assign without ambiguity the two oxidation waves of **[HEL-AMH]+**: the first and second correspond to the oxidation of the dimethylamino group (reaction (1)) and of the diaza core (reaction (3)), respectively. In acetonitrile, the oxidation of helicene [4]diaza complexes is reversible and it means that the resulting electrogenerated cation is stable in such an organic solvent. In PBS, the oxidation wave of the diaza core is irreversible, indicating that the oxidized radical of the diaza core is not stable over the timescale of the voltammetric experiments. In addition, since the same oxidation potential of the diaza core is obtained with and without the dimethylamino group, it implies that the electronic communication between these groups is negligible. This is further supported by the near identical oxidation potential of TPrA and the dimethyl amino moiety of **[HEL-AMH]+**. In brief, the two parts of the luminophore-coreactant molecule behave independently from an electrochemical point of view. This decoupling is an essential prerequisite for the ECL mechanism, which involves intermolecular electron-transfer reactions with the organometallic complexes to enable multicolor ECL emission (*vide infra*).

At an applied potential above 1.3 V, both parts of the molecules are oxidized and the resulting trication [HEL˙-AMH˙]^3+^ intermediate is probably formed (reaction (3)) and may deprotonate to form [HEL˙-AM˙]^2+^ (reaction (4)). We can assume that the coreactant mechanism for the amino moiety is analogous to that of the model TPrA reagent (see the detailed structures of the amino radicals in Scheme S1†).^64,65^

1 **[HEL-AMH]**^**+**^ − e^−^ → [HEL-AMH˙]^2+^

2 [HEL-AMH˙]^2+^ → [HEL-AM˙]^+^ + H^+^

3 [HEL-AMH˙]^2+^ − e^−^ → [HEL˙-AMH˙]^3+^

4 [HEL˙-AMH˙]^3+^ → [HEL˙-AM˙]^2+^ + H^+^

Then we investigated the ECL properties of this helicene molecule. As shown on Fig. 2, ECL light is only emitted at potentials higher than 1.2 V. When the potential is scanned towards more positive values, ECL emission grew and reached a maximum at 1.32 V. It means that ECL generation requires explicitly the oxidation of the diaza core. ECL is obtained without adding any external coreactant; the dimethylamino moiety acting by itself as a coreactant in the mechanism. In brief, self-enhanced ECL emission is generated by this helicene compound. We performed a series of control experiments with the analogous [HEL-SO_3_H]^+^ compound and ECL was generated only after adding TPrA. In other words, the cyclic amines of the [4]diaza core cannot act as coreactants to produce self-enhanced ECL. The excited state of the helicene may be reached by intermolecular (reaction (5)) electron-transfer reactions between the strong radical reductant (denoted-AM˙) and the oxidized diaza core (HEL˙). Both inter- and intra-molecular pathways may be postulated and they depend on the radical lifetimes, their reactivity and concentration, amongst other factors. Even if the two sub-units of the **[HEL-AMH]+** molecule are electronically decoupled, we cannot exclude that the excited state is also generated by the intramolecular electron-transfer reaction (reaction (6)) due to the flexibility of the short linker connecting them. The distance between both sub-units might be small enough to favor intramolecular electron-transfer reaction without a strong coupling that will modify their respective redox potentials. Indeed, Bard and co-workers mentioned the possibility of intramolecular ECL in chlorpromazine, which is a tricyclic ring system with a tertiary amine on the side chain.^66^ It is analogous to the present case with the oxidation and ECL of the helicene [4]diaza core with the amine. Moreover, Sun *et al.* reported the intra- and inter-molecular ECL reactions of ruthenium tris-bipyridyl complexes with different amines.^67^ Finally, photoinduced intramolecular electron-transfer reactions have been reported between several classes of luminophores (*e.g.* pyrene, rhodamine) and amino groups connected by a short alkyl linker.^68–71^ In addition, self-enhanced ECL of iridium complexes with different amine reductants has been also described.^45^ Self-enhanced ECL has been also applied for sensing applications.^42,72–74^ These findings support the possibility of the intramolecular electron-transfer reaction in our system. The excited state of the helicene compound then deactivates through the emission of a photon at 650 nm (reactions (7) and (8)). In this pathway, ECL generation requires the simultaneous presence of both radicals to form the excited state. Even if the electrogenerated radicals are not stable during the timescale of the voltammetric experiments, the ECL emission is an indirect proof of their formation at the vicinity of the electrode surface. In the present case, the resulting ECL spectrum is identical to the fluorescence spectrum (Fig. S14†). Therefore, the same excited state is reached by photo-excitation and by self-enhanced ECL. By assuming that the dimethyl amino moiety of **[HEL-AMH]+** exhibits the same potential as TPrA˙ after deprotonation, it is possible to calculate the energy available from the reaction (5) or (6) (Δ*H*° = *E*°_red_ (TPrA˙) − *E*°_ox_ (helicene core) ≈ −3 eV with *E*°_red_ (TPrA˙) being reported to be −1.7 V *vs.* Ag/AgCl^64,75^). A direct comparison with the emission wavelength of the [4]helicene core (*E*_Fl_ = 1239.81/*λ*_max_ = 1.9 eV) indicates that the excited state can be directly populated.

5 2 [HEL˙-AM˙]^2+^ → [HEL*-AM˙]^+^ + [HEL˙-IM]^3+^

6 [HEL˙-AM˙]^2+^ → [HEL*-IM]^2+^

7 [HEL*-IM]^2+^ → [HEL-IM]^2+^ + *hν*_red_ (650 nm)

8 [HEL*-AM˙]^+^ → [HEL-AM˙]^+^ + *hν*_red_ (650 nm)

where IM represents the iminium product (see the detailed structures in Scheme S1†).^64,76^

**Fig. 3:**
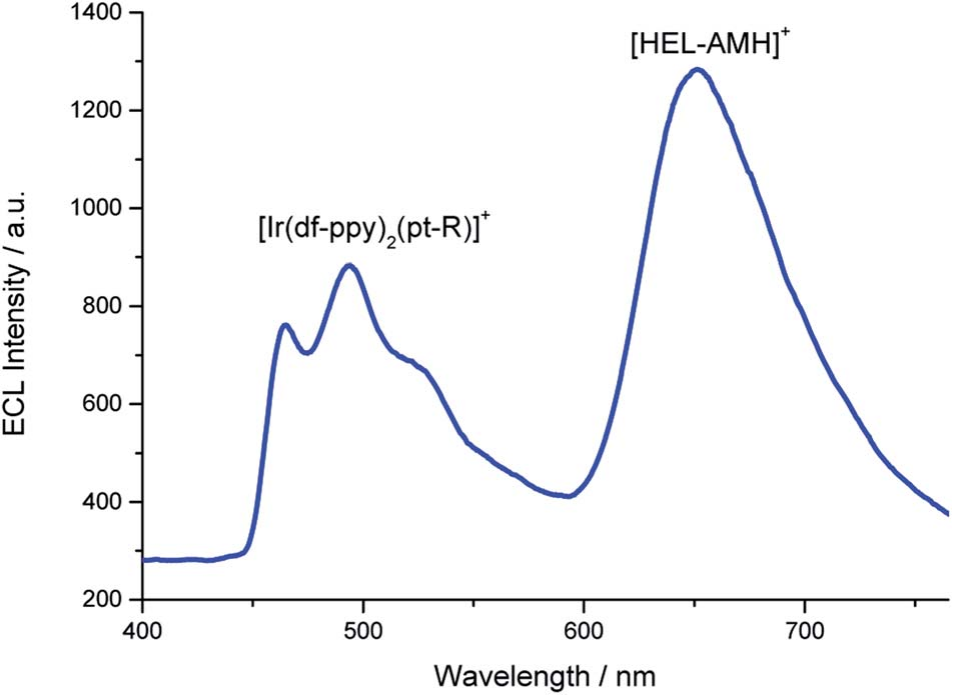
ECL spectrum recorded in a PBS solution containing 10^−4^ M **[HEL-AMH]+** and 10^−3^ M [Ir(df-ppy)_2_(pt-R)]^+^. Applied potential: 1.7 V *vs.* Ag/AgCl/KCl.

Annihilation ECL of [Ru(bpy)_3_]^2+^ with several iridium(iii) complexes has been investigated previously in organic solvents.^28,29^ In the present work, our goal was to generate multicolor ECL emission by combining it with self-enhanced ECL. For this purpose, we added first [Ir(df-ppy)_2_(pt-R)]^+^ (Fig. 1a, where R is a trioxatridecane chain (TOxT) and terminal squarate amide ethyl ester (Sq))^77^ to an aqueous solution containing **[HEL-AMH]+**. The Sq functionality does not change the electrochemical and photophysical properties of the parent luminophore, but provides a convenient attachment point for future works involving biomolecular labelling and multicolor ECL biodetection. Oxidation of the [Ir(df-ppy)_2_(pt-R)]^+^ complex occurs at a higher potential than the diaza core and the corresponding ECL emission is obtained at 1.5 V (Fig. S15†). When both reagents are present, blue and red ECL emissions of [Ir(df-ppy)_2_(pt-R)]^+^ and diaza luminophores are observed concomitantly (Fig. 3).

Such a dual ECL emission is achieved without adding any external coreactant. As already mentioned, FRET processes from the diaza [4]helicene to the iridium dyes are not possible since the emission of [Ir(df-ppy)_2_(pt-R)]^+^ occurs at lower wavelengths than that of the diaza [4]helicene. It is likewise the same energetic configuration for all the three tested organometallic luminophores. The excited states of both luminophores are reached following the reactions occurring between their respective oxidized forms (*i.e.* [Ir(df-ppy)_2_(pt-R)]^2+^ and [HEL˙-AMH]^2+^) and the radical -AM˙ of the modified helicene (reaction (10)). For the iridium complex, it corresponds to an intermolecular electron-transfer reaction with the radical -AM˙ moiety (reaction (10)). The coreactant function of the diaza complex is activated and operates *via* intermolecular mode for [Ir(df-ppy)_2_(pt-R)]^2+^ (reactions (9) and (10)) and inter- and/or intra-molecular modes for **[HEL-AMH]+** (reactions (5) and/or (6)).

9 [Ir(df-ppy)_2_(pt-R)]^+^ − e^−^ → [Ir(df-ppy)_2_(pt-R)]^2+^

10 [Ir(df-ppy)_2_(pt-R)]^2+^ + [HEL˙-AM˙]^2+^ → [Ir(df-ppy)_2_(pt-R)]^+^* + [HEL˙-IM]^3+^

11 [Ir(df-ppy)_2_(pt-R)]^+^* → [Ir(df-ppy)_2_(pt-R)]^+^ + *hν*_blue_

Thus, the **[HEL-AMH]+** molecule, which is a red-emitter, induces also the generation of blue ECL by [Ir(df-ppy)_2_(pt-R)]^+^ (reaction (11)). The final result is reminiscent of an upconversion process, but it is not mechanistically the case here. The blue ECL emission results from a very exergonic electron-transfer reaction from the [HEL˙-AM˙]^2+^ radical to the oxidized [Ir(df-ppy)_2_(pt-R)]^2+^. It is not related to an energy-transfer process such as triplet–triplet annihilation. Herein, we solely control and exploit the dual reactivity of the **[HEL-AMH]+** molecule.

**Fig. 4:**
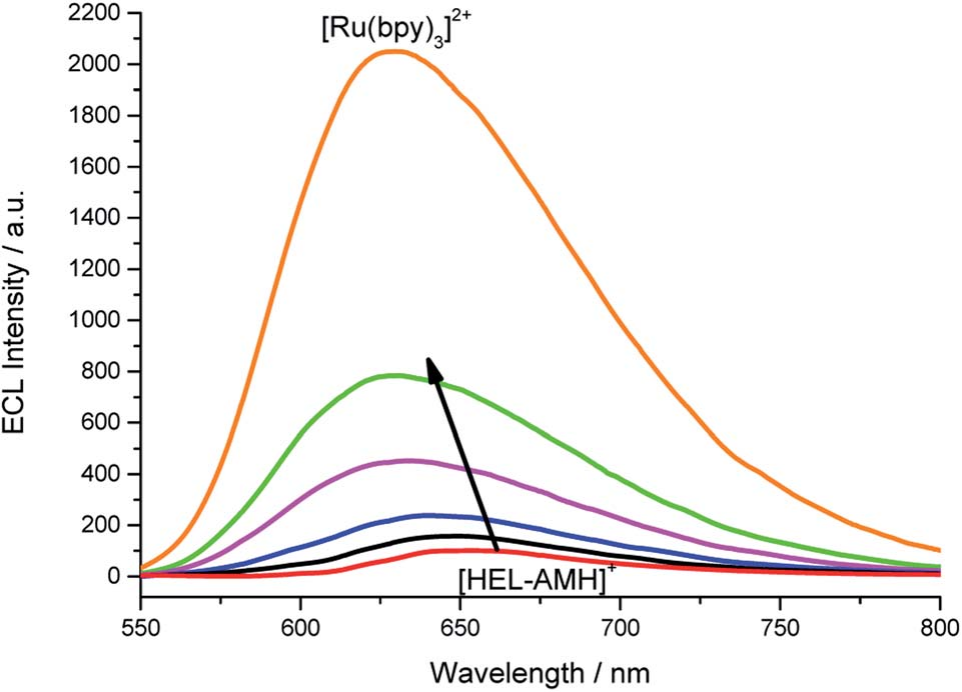
ECL spectra recorded in a PBS solution containing a constant concentration of 10^−4^ M **[HEL-AMH]+** and increasing concentrations of [Ru(bpy)_3_]^2+^: 0, 1 × 10^−5^ M, 3 × 10^−5^ M, 6 × 10^−5^ M, 1 × 10^−4^ M and 1 × 10^−3^ M, as indicated by the arrow. Applied potential: 1.5 V *vs.* Ag/AgCl/KCl.

In the previous example, we showed that ECL can be tuned from red to blue by intermolecular electron-transfer processes. Herein, we investigate the ECL of [Ru(bpy)_3_]^2+^ using this original configuration. Indeed, this complex is the model and most widely used ECL luminophore due to its remarkable stability and high ECL efficiency in water. We tested its behavior by increasing its concentration in a solution containing the **[HEL-AMH]+** compound (Fig. S16†). The maximum ECL intensity moved progressively from 650 nm (characteristic of **[HEL-AMH]+**) to 620 nm (Fig. 4), corresponding to the emission of [Ru(bpy)_3_]^2+^. This shift is even more striking in the corresponding normalized ECL spectra (Fig. S17†). Moreover, one can observe that the [Ru(bpy)_3_]^2+^ ECL band increases remarkably. A similar ECL mechanism as that described above for the iridium luminophore (reactions (9) and (10)) can be proposed.

In a solution containing both luminophores at the same 10^−4^ M concentration (green curve, Fig. 4), light intensity is 8-fold higher in comparison to the solution containing only the **[HEL-AMH]+** (red curve, Fig. 4). When the [Ru(bpy)_3_]^2+^ concentration is increased up to 10^−3^ M, ECL intensity is 20.5-fold higher. We checked that addition of [Ru(bpy)_3_]^2+^ does not influence the fluorescence of the **[HEL-AMH]+** (Fig. S18†). Therefore, the enhancement of the [Ru(bpy)_3_]^2+^ ECL band is due to the very efficient intermolecular electron-transfer between both species and to the high ECL efficiency of the [Ru(bpy)_3_]^2+^ luminophore. Indeed, **[HEL-AMH]+** was found to have an ECL efficiency of 7.3% in comparison to [Ru(bpy)_3_]^2+^ (Table S1†), which accounts for the dramatic increase in ECL intensity. This creates an intriguing situation in which ECL emission resulting from an intermolecular electron-transfer reaction (*i.e.* with [Ru(bpy)_3_]^2+^) is more efficient than that of the intra- and intermolecular processes (*i.e.***[HEL-AMH]+**). The self-enhanced ECL efficiency of **[HEL-AMH]+** with [Ru(bpy)_3_]^2+^ is 8.3-fold higher in comparison to [Ir(df-ppy)_2_(pt-R)]^+^ (Table S1†). This ratio is surprisingly high considering the respective values of the photoluminescence quantum yield and ECL efficiency. It might be related to the remarkable stability of the oxidized form of the ruthenium complex in aqueous media. The oxidation process is irreversible for the iridium complex in aqueous media (see Fig. S15†).

As shown by the voltammetric study, the two moieties of **[HEL-AMH]+** are electronically decoupled to a large extent. This enables the two functions to be operated either separately or concomitantly by controlling the applied potential and the energetics of the luminophores. The coreactant function of **[HEL-AMH]+** is activated at 0.8 V where the dimethylamino group is oxidized. The ECL of this molecule, however, requires oxidation of the diaza core at 1.33 V. Therefore, a wide potential window exists between 0.8 V and 1.33 V where another luminophore can be oxidized and generate ECL before the emission of **[HEL-AMH]+**. For this purpose, we used the green emitter, [Ir(ppy)_2_(pt-R)]^+^ (Fig. 1). Oxidation of this iridium complex occurs at 1.14 V (Fig. S19†), which in the presence of a suitable coreactant elicits green ECL.

**Fig. 5:**
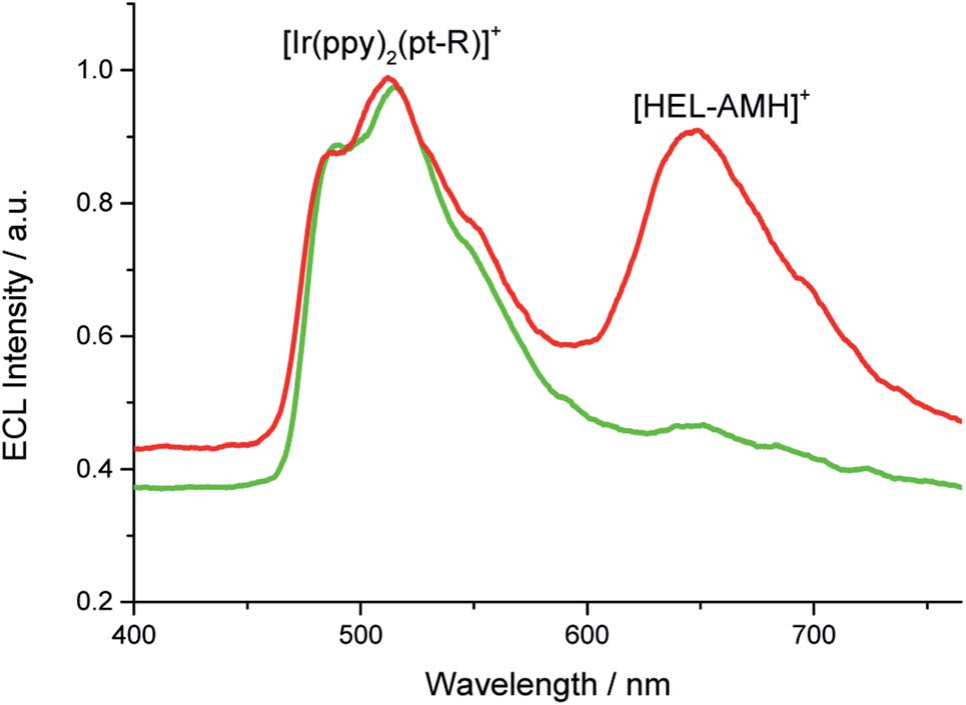
ECL spectra recorded in a PBS solution containing 10^−4^ M **[HEL-AMH]+** and 10^−3^ M [Ir(ppy)_2_(pt-R)]^+^ when applying either 1.1 V (green curve) or 1.3 V (red curve).

By imposing 1.1 V, we observed predominantly the green ECL emitted by the [Ir(ppy)_2_(pt-R)]^+^ and also a small shoulder at 650 nm, which corresponds to the emission wavelength of **[HEL-AMH]+** (green curve, Fig. 5). This potential induces the oxidation of [Ir(ppy)_2_(pt-R)]^+^ as well as of the dimethylamino moiety of **[HEL-AMH]+**, but the diaza core is not oxidized, so the red emission of this luminophore should not be observed (*vide infra*). The oxidized and deprotonated dimethylamino moiety (-AM˙) acts then only as a coreactant by intermolecular electron-transfer with the oxidized [Ir(ppy)_2_(pt-R)]^2+^, as described by the sequence of reactions (1), (2), (12), (13) and (14):

12 [Ir(ppy)_2_(pt-R)]^+^ − e^−^ → [Ir(ppy)_2_(pt-R)]^2+^

13 [Ir(ppy)_2_(pt-R)]^2+^ + [HEL-AM˙]^+^ → [Ir(ppy)_2_(pt-R)]^+^* + [HEL-IM]^2+^

14 [Ir(ppy)_2_(pt-R)]^+^* → [Ir(ppy)_2_(pt-R)]^+^ + *hν*_green_

Under these conditions, only the coreactant function of the helicene molecule is activated and green ECL emission of [Ir(ppy)_2_(pt-R)]^+^ is dominant (green curve, Fig. 5). At higher potentials, the red and the green ECL emissions of **[HEL-AMH]+** and [Ir(ppy)_2_(pt-R)]^+^ are visible concomitantly. The luminescent function of the helicene molecule is activated as well. The helicene molecule acts both as a coreactant and a luminophore. In this potential region, the diaza core of the helicene molecule is also oxidized and it reacts with the -AM˙ moiety radical to give its characteristic red emission. In brief, single or multicolor ECL emission is obtained by controlling the reactivity of the dual **[HEL-AMH]+** luminophore. It is also noteworthy that the resulting color can also be tuned by adjusting the ratio of luminophores dissolved in solution.

Finally, the wide absorption spectrum of the diaza [4]helicene (Fig. S9†) overlays with the emission spectra of the iridium (or ruthenium) dyes (Fig. 1b). Therefore, FRET may occur from the donor organometallic dyes, once “electro-excited” by the radical amino moiety of the helicene, to the diaza core of the helicene. Such an effect is not directly visible with [Ir(df-ppy)_2_(pt-R)]^2+^ or [Ru(bpy)_3_]^2+^ because the potentials required to generate ECL of these organometallic dyes induce also the ECL emission of the helicene. The respective contributions cannot be easily deconvoluted. But, the energetic configuration is different with [Ir(ppy)_2_(pt-R)]^+^. As already mentioned for this compound, when imposing 1.1 V, a small shoulder is observed on Fig. 5 (green curve) at the emission wavelength of the **[HEL-AMH]+**. Since the diaza core is not oxidized at this potential and should not thus emit ECL, it is possible that it corresponds to the emission of the helicene by FRET. It would then correspond to an appealing “circular pattern” where the electron-transfer from the electron donor helicene to the electron-acceptor iridium dye may then lead to the FRET from the energy-donor iridium dye to the energy-acceptor diaza [4]helicene. Essentially, the intermolecular electron-transfer in one direction may result in back energy-transfer in the opposite direction. However, we cannot exclude completely the possibility that a very small fraction of **[HEL-AMH]+** is oxidized at this potential due to the proximity of both potential values. Although it might be difficult to decipher the different contributions in such a complex mechanistic schemes, we plan to investigate such possibilities in future works.

## Conclusions

Self-enhanced ECL emission is demonstrated in aqueous media by integrating bifunctional ECL features in a single molecular system using a red-emitting diaza [4]helicene functionalized with a dimethylamino moiety. The coreactant and luminescent functions are decoupled and can be activated either separately or simultaneously by controlling the applied potential. Self-enhanced multicolor ECL, from red to blue, *via* orange and green, is obtained by competitive electron-transfer reactions between the helicene and a series of organometallic iridium or ruthenium-based complexes. Single or multicolor emissions with tunable intensity are produced by controlling the dual reactivity of the helicene molecule. This multicolor ECL strategy can be extended to other luminophores and coreactants and has important implications for electron-transfer and energy-transfer processes, multiplexed bioassays and imaging applications.

## Conflicts of interest

There are no conflicts to declare.

## Supplementary Material

SC-011-D0SC00853B-s001

## References

[cit1] Bard A. J. and Faulkner L. R., Electrochemical methods, Wiley, New York, 2001

[cit2] Bard A. J., Electrogenerated Chemiluminescence, M. Dekker, New-York, 2004

[cit3] Liu Z., Qi W., Xu G. (2015). Chem. Soc. Rev..

[cit4] Miao W. (2008). Chem. Rev..

[cit5] Sojic N., Analytical Electrogenerated Chemiluminescence: From Fundamentals to Bioassays, Royal Society of Chemistry (RSC) Publishing, 2020

[cit6] Schmittel M., Lin H.-W. (2007). Angew. Chem., Int. Ed..

[cit7] Irkham I., Watanabe T., Fiorani A., Valenti G., Paolucci F., Einaga Y. (2016). J. Am. Chem. Soc..

[cit8] Irkham I., Fiorani A., Valenti G., Kamoshida N., Paolucci F., Einaga Y. (2020). J. Am. Chem. Soc..

[cit9] Yuan Y., Han S., Hu L., Parveen S., Xu G. (2012). Electrochim. Acta.

[cit10] Liu X., Shi L., Niu W., Li H., Xu G. (2007). Angew. Chem., Int. Ed..

[cit11] Dinel M.-P., Tartaggia S., Wallace G. Q., Boudreau D., Masson J.-F., Polo F. (2019). Angew. Chem., Int. Ed..

[cit12] Zinna F., Voci S., Arrico L., Brun E., Homberg A., Bouffier L., Funaioli T., Lacour J., Sojic N., Di Bari L. (2019). Angew. Chem., Int. Ed..

[cit13] AlTal F., Gao J. (2018). J. Am. Chem. Soc..

[cit14] Gao J. (2018). Curr. Opin. Electrochem..

[cit15] Zhao Y., Yu J., Xu G., Sojic N., Loget G. (2019). J. Am. Chem. Soc..

[cit16] Collinson M. M., Pastore P., Maness K. M., Wightman R. M. (1994). J. Am. Chem. Soc..

[cit17] Xu L., Li Y., Wu S., Liu X., Su B. (2012). Angew. Chem., Int. Ed..

[cit18] Fan F.-R. F., Bard A. J. (2008). Nano Lett..

[cit19] Dick J. E., Renault C., Kim B.-K., Bard A. J. (2014). Angew. Chem., Int. Ed..

[cit20] Zhu M.-J., Pan J.-B., Wu Z.-Q., Gao X.-Y., Zhao W., Xia X.-H., Xu J.-J., Chen H.-Y. (2018). Angew. Chem., Int. Ed..

[cit21] Ma C., Wu W., Li L., Wu S., Zhang J., Chen Z., Zhu J.-J. (2018). Chem. Sci..

[cit22] Chen S., Ma H., Padelford J. W., Qinchen W., Yu W., Wang S., Zhu M., Wang G. (2019). J. Am. Chem. Soc..

[cit23] Voci S., Goudeau B., Valenti G., Lesch A., Jović M., Rapino S., Paolucci F., Arbault S., Sojic N. (2018). J. Am. Chem. Soc..

[cit24] Ding H., Guo W., Su B. (2020). Angew. Chem., Int. Ed..

[cit25] Valenti G., Scarabino S., Goudeau B., Lesch A., Jović M., Villani E., Sentic M., Rapino S., Arbault S., Paolucci F., Sojic N. (2017). J. Am. Chem. Soc..

[cit26] Han F., Jiang H., Fang D., Jiang D. (2014). Anal. Chem..

[cit27] Zhang J., Jin R., Jiang D., Chen H.-Y. (2019). J. Am. Chem. Soc..

[cit28] Doeven E. H., Zammit E. M., Barbante G. J., Hogan C. F., Barnett N. W., Francis P. S. (2012). Angew. Chem., Int. Ed..

[cit29] Doeven E. H., Zammit E. M., Barbante G. J., Francis P. S., Barnett N. W., Hogan C. F. (2013). Chem. Sci..

[cit30] Kerr E., Doeven E. H., Barbante G. J., Hogan C. F., Bower D. J., Donnelly P. S., Connell T. U., Francis P. S. (2015). Chem. Sci..

[cit31] Zanarini S., Felici M., Valenti G., Marcaccio M., Prodi L., Bonacchi S., Contreras-Carballada P., Williams R. M., Feiters M. C., Nolte R. J. M., De Cola L., Paolucci F. (2011). Chem. - Eur. J..

[cit32] Schmittel M., Shu Q., Cinar M. E. (2012). Dalton Trans..

[cit33] Swanick K. N., Ladouceur S., Zysman-Colman E., Ding Z. (2012). Chem. Commun..

[cit34] Kapturkiewicz A. (2016). Anal. Bioanal. Chem..

[cit35] Kapturkiewicz A., Angulo G. (2003). Dalton Trans..

[cit36] Zanarini S., Rampazzo E., Bonacchi S., Juris R., Marcaccio M., Montalti M., Paolucci F., Prodi L. (2009). J. Am. Chem. Soc..

[cit37] Muegge B. D., Richter M. M. (2004). Anal. Chem..

[cit38] Kapturkiewicz A., Chen T.-M., Laskar I. R., Nowacki J. (2004). Electrochem. Commun..

[cit39] Valenti G., Rampazzo E., Bonacchi S., Khajvand T., Juris R., Montalti M., Marcaccio M., Paolucci F., Prodi L. (2012). Chem. Commun..

[cit40] Rizzo F., Polo F., Bottaro G., Fantacci S., Antonello S., Armelao L., Quici S., Maran F. (2017). J. Am. Chem. Soc..

[cit41] Li H., Bouffier L., Arbault S., Kuhn A., Hogan C. F., Sojic N. (2017). Electrochem. Commun..

[cit42] Guo W., Ding H., Gu C., Liu Y., Jiang X., Su B., Shao Y. (2018). J. Am. Chem. Soc..

[cit43] Soulsby L. C., Hayne D. J., Doeven E. H., Wilson D. J. D., Agugiaro J., Connell T. U., Chen L., Hogan C. F., Kerr E., Adcock J. L., Donnelly P. S., White J. M., Francis P. S. (2018). Phys. Chem. Chem. Phys..

[cit44] Soulsby L. C., Doeven E. H., Pham T. T., Eyckens D. J., Henderson L. C., Long B. M., Guijt R. M., Francis P. S. (2019). Chem. Commun..

[cit45] Swanick K. N., Ladouceur S., Zysman-Colman E., Ding Z. (2012). Angew. Chem., Int. Ed..

[cit46] Hesari M., Swanick K. N., Lu J.-S., Whyte R., Wang S., Ding Z. (2015). J. Am. Chem. Soc..

[cit47] Omer K. M., Ku S.-Y., Cheng J.-Z., Chou S.-H., Wong K.-T., Bard A. J. (2011). J. Am. Chem. Soc..

[cit48] Polo F., Rizzo F., Veiga-Gutierrez M., De Cola L., Quici S. (2012). J. Am. Chem. Soc..

[cit49] Li H., Daniel J., Verlhac J.-B., Blanchard-Desce M., Sojic N. (2016). Chem. - Eur. J..

[cit50] Shen M., Zhu X.-H., Bard A. J. (2013). J. Am. Chem. Soc..

[cit51] Adam C., Wallabregue A., Li H., Gouin J., Vanel R., Grass S., Bosson J., Bouffier L., Lacour J., Sojic N. (2015). Chem. - Eur. J..

[cit52] Oh J.-W., Lee Y. O., Kim T. H., Ko K. C., Lee J. Y., Kim H., Kim J. S. (2009). Angew. Chem., Int. Ed..

[cit53] Fleet B., Kirkbright G. F., Pickford C. J. (1971). J. Electroanal. Chem. Interfacial Electrochem..

[cit54] Kanibolotsky A. L., Laurand N., Dawson M. D., Turnbull G. A., Samuel I. D. W., Skabara P. J. (2019). Acc. Chem. Res..

[cit55] Shen Y., Chen C.-F. (2012). Chem. Rev..

[cit56] Bosson J., Gouin J., Lacour J. (2014). Chem. Soc. Rev..

[cit57] Hernández Delgado I., Pascal S., Wallabregue A., Duwald R., Besnard C., Guénée L., Nançoz C., Vauthey E., Tovar R. C., Lunkley J. L., Muller G., Lacour J. (2016). Chem. Sci..

[cit58] Babič A., Pascal S., Duwald R., Moreau D., Lacour J., Allémann E. (2017). Adv. Funct. Mater..

[cit59] Duwald R., Pascal S., Bosson J., Grass S., Besnard C., Bürgi T., Lacour J. (2017). Chem. - Eur. J..

[cit60] Bauer C., Duwald R., Labrador G. M., Pascal S., Moneva Lorente P., Bosson J., Lacour J., Rochaix J.-D. (2018). Org. Biomol. Chem..

[cit61] Duwald R., Bosson J., Pascal S., Grass S., Zinna F., Besnard C., Di Bari L., Jacquemin D., Lacour J. (2020). Chem. Sci..

[cit62] Li H., Wallabregue A., Adam C., Labrador G. M., Bosson J., Bouffier L., Lacour J., Sojic N. (2017). J. Phys. Chem. C.

[cit63] Pascal S., Besnard C., Zinna F., Di Bari L., Le Guennic B., Jacquemin D., Lacour J. (2016). Org. Biomol. Chem..

[cit64] Miao W., Choi J.-P., Bard A. J. (2002). J. Am. Chem. Soc..

[cit65] Sentic M., Milutinovic M., Kanoufi F., Manojlovic D., Arbault S., Sojic N. (2014). Chem. Sci..

[cit66] Richards T. C., Bard A. J. (1995). Anal. Chem..

[cit67] Sun S., Yang Y., Liu F., Fan J., Peng X., Kehr J., Sun L. (2009). Dalton Trans..

[cit68] Aigner D., Freunberger S. A., Wilkening M., Saf R., Borisov S. M., Klimant I. (2014). Anal. Chem..

[cit69] Escudero D. (2016). Acc. Chem. Res..

[cit70] Swinnen A. M., Van der Auweraer M., De Schryver F. C., Nakatani K., Okada T., Mataga N. (1987). J. Am. Chem. Soc..

[cit71] Okada T., Migita M., Mataga N., Sakata Y., Misumi S. (1981). J. Am. Chem. Soc..

[cit72] Wang H., Yuan Y., Zhuo Y., Chai Y., Yuan R. (2016). Anal. Chem..

[cit73] Yang L., Zhang B., Fu L., Fu K., Zou G. (2019). Angew. Chem., Int. Ed..

[cit74] Shu J., Han Z., Zheng T., Du D., Zou G., Cui H. (2017). Anal. Chem..

[cit75] Stringer B. D., Quan L. M., Barnard P. J., Wilson D. J. D., Hogan C. F. (2014). Organometallics.

[cit76] Qiu R., Zhang X., Luo H., Shao Y. (2016). Chem. Sci..

[cit77] Chen L., Hayne D. J., Doeven E. H., Agugiaro J., Wilson D. J. D., Henderson L. C., Connell T. U., Nai Y. H., Alexander R., Carrara S., Hogan C. F., Donnelly P. S., Francis P. S. (2019). Chem. Sci..

