# Supplementary material for: Self-enhanced multicolor electrochemiluminescence by competitive electron-transfer processes
Source: Chem Sci. 2020 Apr 17;11(17):4508–15. doi: 10.1039/d0sc00853b (PMC8159437; doi:10.1039/d0sc00853b)
Supplement: SC-011-D0SC00853B-s001 [file SC-011-D0SC00853B-s001.pdf]

Electronic Supplementary Material (ESI) for Chemical Science.
This journal is © The Royal Society of Chemistry 2020

Electronic Supporting Information

Self-Enhanced Multicolor Electrochemiluminescence by Competitive Electron-transfer Processes

Silvia Voci,[a] Romain Duwald,[b] Stéphane Grass,[b] David J. Hayne,[c] Laurent Bouffier,[a] Paul S. Francis,[c] Jérôme Lacour,[b] Neso Sojic[a*]

[a]Univ. Bordeaux, Bordeaux INP, CNRS. Institut des Sciences Moléculaires, UMR 5255. 33607 Pessac (France). E-mail: neso.sojic@enscbp.fr

[b]University of Geneva, Department of Organic Chemistry, Quai Ernest Ansermet 30, 1211 Geneva 4 (Switzerland).

[c]Deakin University, School of Life and Environmental Sciences, Faculty of Science, Engineering and Built Environment; Waurn Ponds, Victoria 3216 (Australia).

Table of Contents

1. General remarks and analysis conditions S2
2. Synthesis and characterization of $[HEL\text{-}AMH]^+$ and $[HEL\text{-}SO_3H]^+$ S4
3. Water solubility study of $[HEL\text{-}AMH]^+$ and $[HEL\text{-}SO_3H]^+$ S12
4. Electrochemistry and ECL S14
5. Spectroscopic, electrochemical and ECL properties of the dyes S14
6. Mechanism of oxidation/dissociation of the tertiary amine moiety S15
7. Voltammetric characterization of the [4]diaza helicene $[HEL\text{-}SO_3H]^+$ S16
8. Comparison of the fluorescence and ECL spectra of $[HEL\text{-}AMH]^+$ S16
9. Voltammetric and ECL responses of $[HEL\text{-}AMH]^+$ in presence of $[Ir(df\text{-}ppy)_2(pt\text{-}R)]^+$ S17
10. ECL and fluorescence of $[HEL\text{-}AMH]^+$ in presence of $[Ru(bpy)_3]^{2+}$ S18
11. Voltammetric and ECL responses of $[HEL\text{-}AMH]^+$ in presence of $[Ir(ppy)_2(pt\text{-}R)]^+$ S20
10. References S20

1. General remarks and analysis conditions

Abbreviations.

- $[HEL\text{-}AMH]^+$ (red emitter/coreactant): [4]diaza helicene cation with dimethylamine moiety (see Figure 1a in main document)
- $[HEL\text{-}SO_3H]^+$: [4]diaza helicene cation with sulfonate moiety
- HEL: diaza core of the [4]diaza helicene luminophore
- AMH: dimethylamino moiety of the [4]diaza helicene luminophore
- $[Ir(df\text{-}ppy)_2(pt\text{-}R)]^+$ (blue emitter): $[Ir(dF\text{-}ppy)_2(pt\text{-}TOxT\text{-}Sq)]^+$ complex (see Figure 1a)
- $[Ir(ppy)_2(pt\text{-}R)]^+$ (green emitter): $[Ir(ppy)_2(pt\text{-}TOxT\text{-}Sq)]^+$ complex (see Figure 1a)

Reagents

The cationic [4]helicene carboxylic acid precursor was prepared according to reported procedures.[1, 2] Reagents were used as purchased, unless otherwise stated. Reactions were conducted under N_2 atmosphere using standard schlenck technics, unless otherwise stated. Column chromatography were performed using Siliaflash P60 silica gel (40-63 µm, 60 Å), Acros Brockmann I basic alumina (40-200 µm, 60 Å), or were done with a Combi*Flash*® Rf 200 on SiO_2 4 g cartridge. Optical properties were recorded in analytical grade solvents. Preparative TLC were performed using TLC Silica gel 60 F254 plates purchased from Merck.

Analytical methods and apparatus

NMR spectra were recorded on Brucker Advance II+ AMX-500 and AMX-400 spectrometers at room temperature (otherwise noted). NMR chemical shifts are given in ppm (δ) relative to Me_4Si with solvent resonances used as internal standards (CD_2Cl_2: 5.32 ppm for ^1H and 53.84 for ^{13}C; CD_3OD: 3.31 ppm for ^1H and 49.0 for ^{13}C; DMSO-d_6: 2.50 ppm for ^1H and 39.5 for ^{13}C). **IR spectra** were recorded on a Perkin-Elmer 1650 FT-IR spectrometer using a diamond ATR Golden Gate sampling. **Melting points** (M.P.) were measured in open capillary tubes with a Buchi B-550 melting points apparatus and are uncorrected. **R*f*** were measured on TLC Silica gel 60 F254 plates purchased from Merck. **Electrospray mass spectra** were obtained on a Finnigan SSQ 7000 spectrometer QSTAR pulsar *i* (AB / MDS Sciex), ESI (TIS)/nanoESI/APCI-QqTof by the Department of Mass Spectroscopy of the University of Geneva. Optical rotation were measured on a Perkin Elmer 241 polarimeter at 20°C using a Hg lamp (365 nm).

Optical properties

UV-Vis-NIR absorption spectra were recorded on a JASCO V-650 spectrophotometer at 20°C. Measurement were performed in acetonitrile analytical grade at precise concentrations *ca*. 1×10^{-5} M. **Steady-state fluorescence spectra** were measured using a Jobin Yvon Fluoromax-3 spectrofluorimeter in acetonitrile for the helical azaacenes. All fluorescence spectra were corrected for the wavelength-dependent sensitivity of the detection. Fluorescence quantum yields Φ were measured in diluted solution with an optical density lower than 0.1 using the following equation:

$$\frac{\Phi_x}{\Phi_r} = \left(\frac{A_r(\lambda)}{A_x(\lambda)}\right)\left(\frac{n_x^2}{n_r^2}\right)\left(\frac{D_x}{D_r}\right)$$

where A is the absorbance at the excitation wavelength (λ), n the refractive index and D the integrated intensity. “r” and “x” stand for reference and sample. The fluorescence quantum yields were measured relative to cresyl violet in methanol in ethanol (Φ = 0.54). Excitations of reference and sample compounds were performed at the same wavelength.

2. Synthesis and characterization of [HEL-AMH]$^+$ and [HEL-SO_3H]$^+$

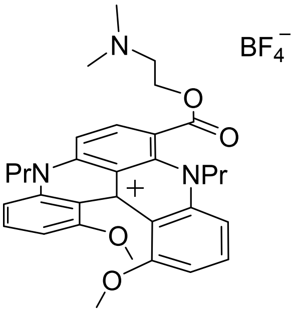

1,13-dimethoxy-5,9-dipropyl-5,9-dihydroquinolino[2,3,4-kl]acridin-13b-ylium-6-carboxylic acid tetrafluoroborate precursor (50 mg, 0.09 mmol, 1 eq.) was dissolved in 2 mL of anhydrous dichloromethane. To this solution was added oxalyl chloride (40 µL, 0.46 mmol, 5 eq.) and the reaction was stirred for 1 hour at room temperature. Then the reaction mixture was cooled to 0°C then freshly distilled 2-(dimethylamino)ethanol (92 µL, 0.92 mmol, 10 eq.) was added. After 10 minutes of stirring at room temperature, the mixture was quenched with water. The organic layer was extracted and washed twice with an aqueous diluted solution of aqueous 1 M HBF_4, dried over Na_2SO_4, filtered and finally evaporated. Purification by silica gel column chromatography using DCM/methanol (95/5, R_f = 0.44) afforded the pure compound [HEL-AMH][BF_4] salt as a dark green solid (42 mg, 74%).

M. P.: 191°C.

^{1}H NMR (CD_2Cl_2, 500 MHz) δ = 8.65 (d, *J* = 9.1 Hz, 1H), 8.03 (dd, *J* = 8.9, 8.1 Hz, 1H), 7.91 (t, *J* = 8.4 Hz, 1H), 7.53 (d, *J* = 9.2 Hz, 1H), 7.51 (d, *J* = 8.7 Hz, 1H), 7.46 (dd, *J* = 8.8, 0.8 Hz, 1H), 7.00 (d, *J* = 8.1 Hz, 1H), 6.92 (dd, *J* = 8.2, 0.8 Hz, 1H), 5.10 – 5.04 (m, 1H), 4.79 – 4.68 (m, 1H), 4.59 – 4.53 (m, 3H), 3.93 – 3.85 (m, 1H), 3.81 (s, 3H), 3.77 (s, 3H), 2.82 (td, *J* = 5.1, 1.6 Hz, 2H), 2.41 (s, 6H), 2.27 – 2.14 (m, 2H), 1.80 – 1.66 (m, 2H), 1.27 (t, *J* = 7.4 Hz, 3H), 0.43 (t, *J* = 7.3 Hz, 3H).

^{13}C NMR (CD_2Cl_2, 126 MHz) δ = 66.0, 160.2, 159.6, 142.3, 141.9, 141.6, 141.4, 140.5, 139.9, 138.6, 137.2, 120.7, 116.7, 114.2, 112.8, 110.6, 107.9, 106.1, 104.8, 104.3, 70.8, 63.4, 59.3, 58.3, 56.5, 56.3, 53.0, 45.8, 22.8, 21.0, 11.4, 10.9.

^{19}F NMR (282 MHz, CD_2Cl_2) δ =-152.98, -153.03.

UV-vis: λ_{max} (CH_3CN) = 592 nm (ε = 11 700 $L.mol^{-1}.cm^{-1}$), λ_{max} (PBS) = 584 nm (ε = 11 600 $L.mol^{-1}.cm^{-1}$).

Fluorescence: λ_{em} (CH_3CN) = 647 nm (ϕ = 22% relative to cresyl violet, 54% in MeOH), λ_{em} (PBS) = 642 nm (ϕ = 21% relative to cresyl violet, 54% in MeOH).

IR (neat, cm^{-1}): ν = 3555, 2944, 2879, 2775, 1711, 1582, 1505, 1467, 1352, 1274, 1238, 1170, 1125, 1045, 817.

HRMS (ESI+) calculated for [M+]: ($C_{32}H_{38}N_3O_4$) 528.2857, Found 528.2869.

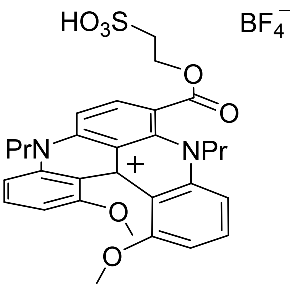

1,13-dimethoxy-5,9-dipropyl-5,9-dihydroquinolino[2,3,4-kl]acridin-13b-ylium-6-carboxylic acid tetrafluoroborate precursor (50 mg, 0.09 mmol, 1 eq.) was dissolved in 3 mL of anhydrous dichloromethane. To this solution was added $SOCl_2$ (34 µL, 0.46 mmol, 5 eq.) and the reaction was stirred for 10 minutes at room temperature. Then 2-hydroxyethane-1-sulfonic acid (116 mg, 0.92 mmol, 10 eq.) was added in 2 mL of anhydrous dichloromethane and after 1 hour of stirring at room temperature the mixture was quenched with water. The organic layer was extracted and washed two times with an aqueous diluted solution of aqueous 1 M HBF_4, dried over Na_2SO_4, filtered and finally evaporated. Purification by silica gel column chromatography using DCM/methanol (90/10, R_f = 0.45) afforded the pure product [HEL-SO_3H][BF_4] salt as a dark green solid (46 mg, 79 %).

M. P.: 200°C.

^{1}H NMR (CD_2Cl_2, 500 MHz) δ = 1H NMR (500 MHz, CD_2Cl_2) δ 7.96 (t, *J* = 8.2 Hz, 1H), 7.89, (t, *J* = 8.3 Hz, 1H), 7.59 (d, *J* = 8.9 Hz, 1H) 7.53 (d, *J* = 9.1 Hz, 1H), 7.44 (d, *J* = 8.9 Hz, 1H), 6.94 (d, *J* = 8.1 Hz, 1H), 6.87 (t, *J* = 7.9 Hz, 1H), 5.16 (dt, *J* = 14.4 Hz, *J* = 5.2 Hz, 1H), 4.93-4.88 (m, 1H), 4.77-4.72 (m, 1H), 4.68-4.62 (m, 1H), 4.51-4.45 (m, 1H), 3.96-3.90 (m, 1H), 3.78 (s, 3H), 3.73 (s, 3H), 3.28-3.19 (m, 2H), 2.24-2.09 (m, 2H), 1.79 (bs, 1H), 1.76-1.69 (m, 2H), 1.24 (t, *J* = 7.6 Hz, 3H), 0.36 (t, *J* = 7.6 Hz, 3H).

^{13}C NMR (CD_2Cl_2, 125 MHz) δ = 13C NMR (126 MHz, CD_2Cl_2) δ 166.3, 160.3, 159.3, 142.4, 141.9, 141.3, 141.3, 140.8, 140.3, 138.1, 137.1, 120.6, 116.6, 114.0, 113.6, 111.4, 107.9, 106.1, 104.4, 104.1, 63.44, 59.4, 56.4, 56.2, 53.0, 50.6, 22.8, 20.9, 11.4, 10.8.

^{19}F NMR (282 MHz, CD_2Cl_2) δ = -153.24, -153.30.

UV-vis: λ_{max} (CH_3CN) = 590 nm (ε = 5 500 L.mol^{-1}.cm^{-1}), λ_{max} (PBS) = 588 nm (ε = 5 200 L.mol^{-1}.cm^{-1}).

Fluorescence: λ_{em} (CH_3CN) = 644 nm (ϕ = 38% relative to cresyl violet, 54% in MeOH), λ_{em} (PBS) = 644 nm (ϕ = 19% relative to cresyl violet, 54% in MeOH).

IR (neat, cm^{-1}): ν = 3444, 2963, 1710, 1595, 1508, 1470, 1274, 1236, 1171, 1126, 1038.

HRMS (ESI+) calculated for [M+]: ($C_{30}H_{33}N_2O_7S$), 565.2003 Found 565.2024.

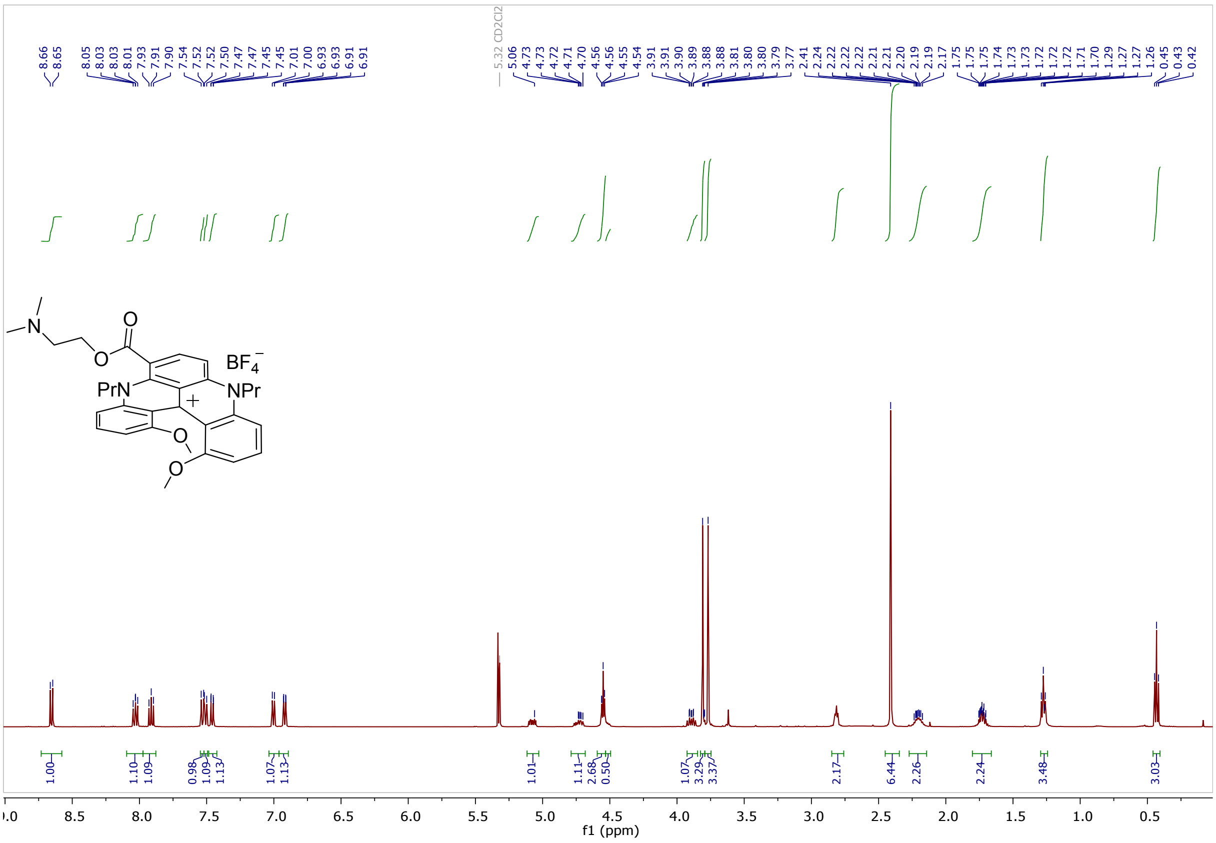

*Figure S1. [1]**H NMR, CD_2Cl_2, 500 MHz** of [HEL-AMH][BF_4]*

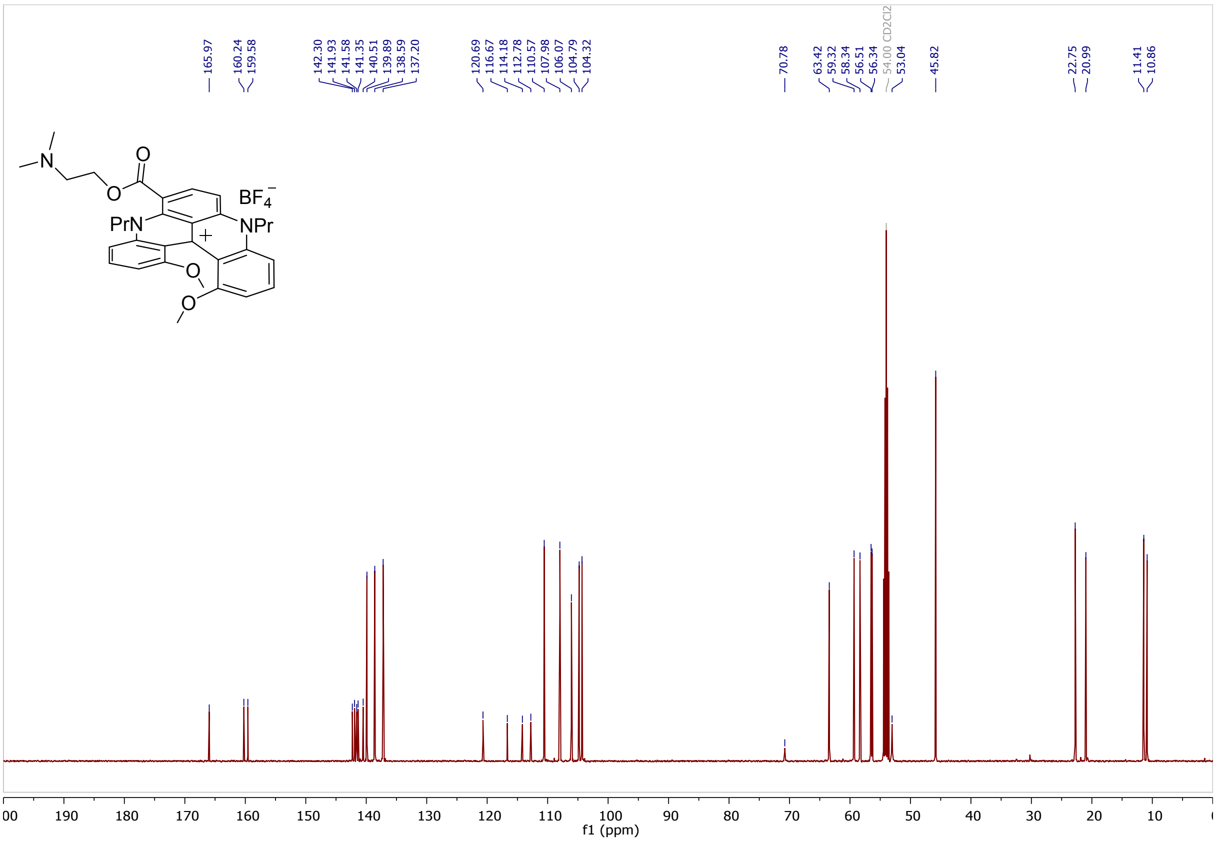

*Figure S2. [13]**C NMR, CD_2Cl_2, 126 MHz** of [HEL-AMH][BF_4]*

Mass Spectrometry Core Facility

Faculty of Sciences – University of Geneva

UNIVERSITÉ DE GENÈVE
FACULTÉ DES SCIENCES

ESI-HRMS – Certificate of Analysis

Applicant:	Romain Duwald	Date of reception:	December 03rd, 2018
Group/Company:	Jérôme Lacour	Date of certificate:	December 05th, 2018
Sample name:	DuR 1099	Data filename:	SMS10GE-181205-HT-A003
Sample number:	9617	Instrument:	QSTAR Pulsar (QqTOF)
**Analyst:**	**Harry Théraulaz**	Ionisation mode:	ESI (positive polarity)
**Contact:**	**esi-hrms@unige.ch**		

Elemental Formula	Ion type	Calc. *m/z*	Exp. *m/z*	Accuracy [a] *(ppm)*
$C_{32}H_{38}N_3O_4$	$[M]^+$	528.2857	528.2869	2.3

[a] *Mass accuracy is determined after spectrum re-calibration (internal calibration with standards added to the FIA mobile phase)*

Recalibrated mass spectrum

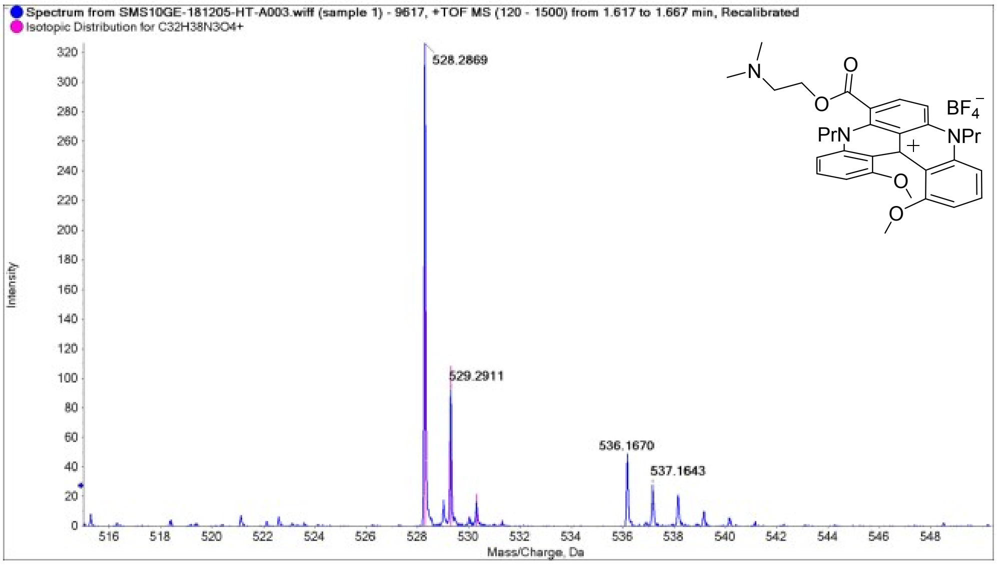

1/1

Figure S3. HRMS certificate of analysis of [HEL-AMH][BF$_4$]

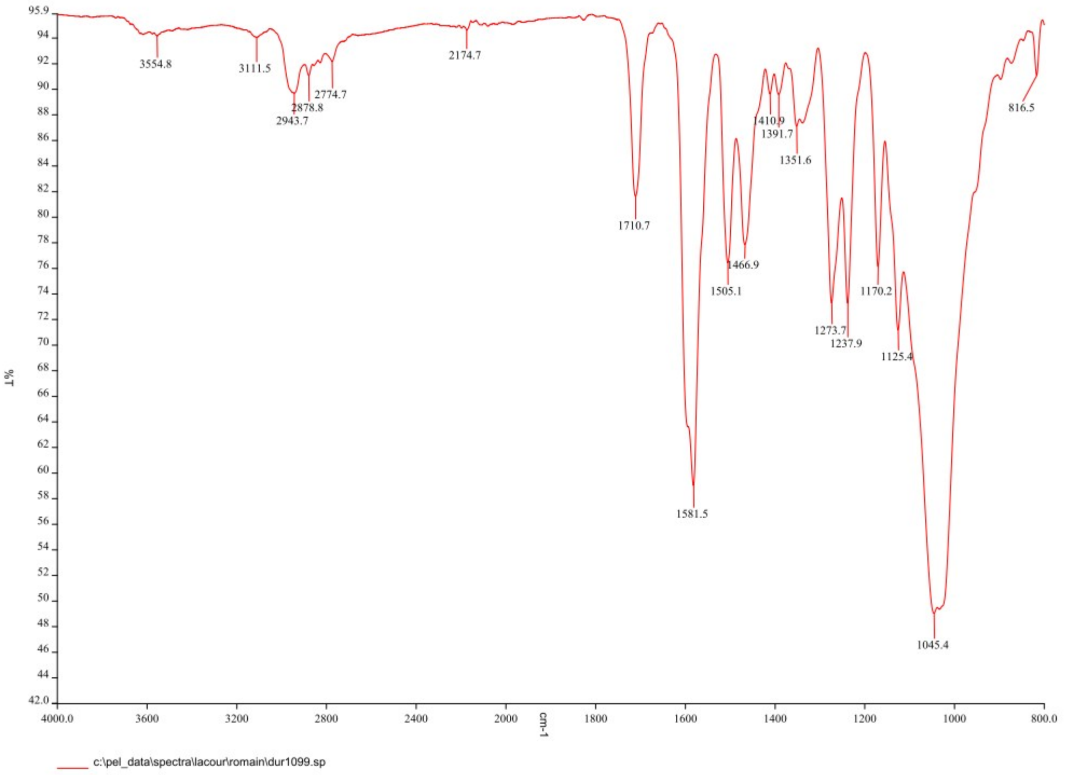

Figure S4. FT-IR analysis of [HEL-AMH][BF_4]

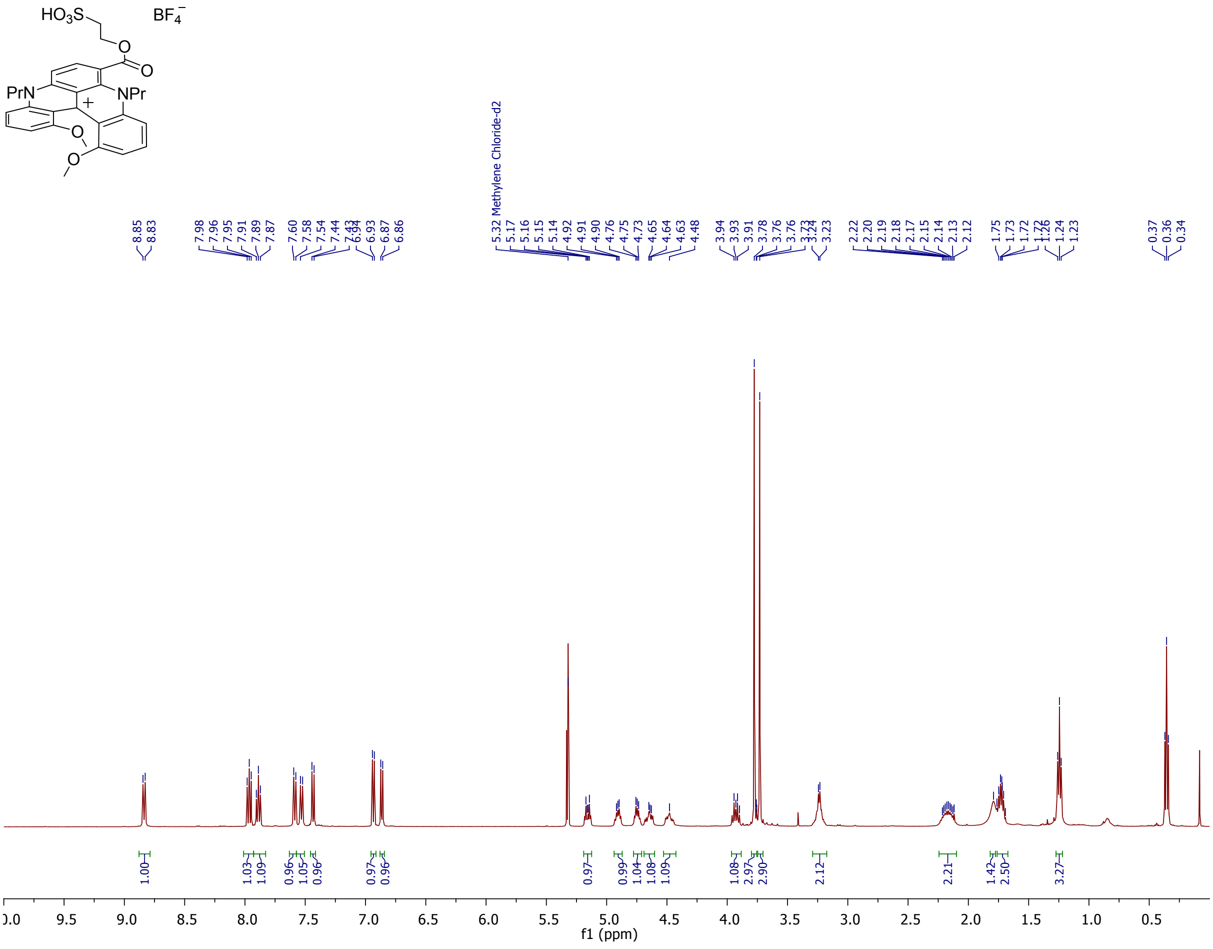

Figure S5 ***^{1}H NMR, CD_2Cl_2, 500 MHz*** *of [HEL-SO_3H][BF_4]*

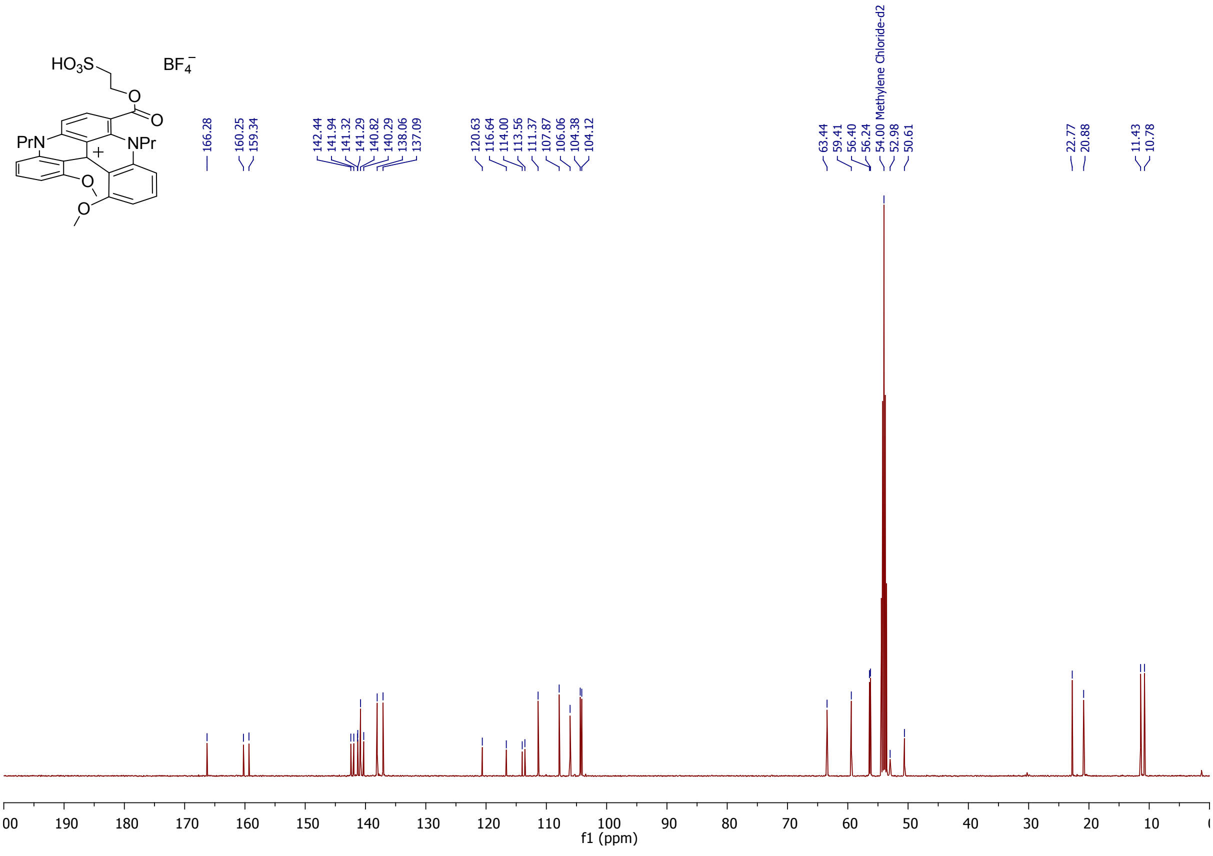

Figure S6. ***^{13}C NMR, CD_2Cl_2, 125 MHz*** *of [HEL-SO_3H][BF_4]*

Mass Spectrometry Core Facility

Faculty of Sciences – University of Geneva

UNIVERSITÉ DE GENÈVE
FACULTÉ DES SCIENCES

ESI-HRMS – Certificate of Analysis

Applicant:	Romain Duwald	Date of reception:	December 03rd, 2018
Group/Company:	Jérôme Lacour	Date of certificate:	December 05th, 2018
Sample name:	DuR 1101	Data filename:	SMS10GE-181205-HT-A004
Sample number:	9619	Instrument:	QSTAR Pulsar (QqTOF)
**Analyst:**	**Harry Théraulaz**	Ionisation mode:	ESI (positive polarity)
**Contact:**	**esi-hrms@unige.ch**		

Elemental Formula	Ion type	Calc. *m/z*	Exp. *m/z*	Accuracy [a] *(ppm)*
$C_{30}H_{33}N_2O_7S$	$[M]^+$	565.2003	565.2024	3.7

[a] *Mass accuracy is determined after spectrum re-calibration (internal calibration with standards added to the FIA mobile phase)*

Recalibrated mass spectrum

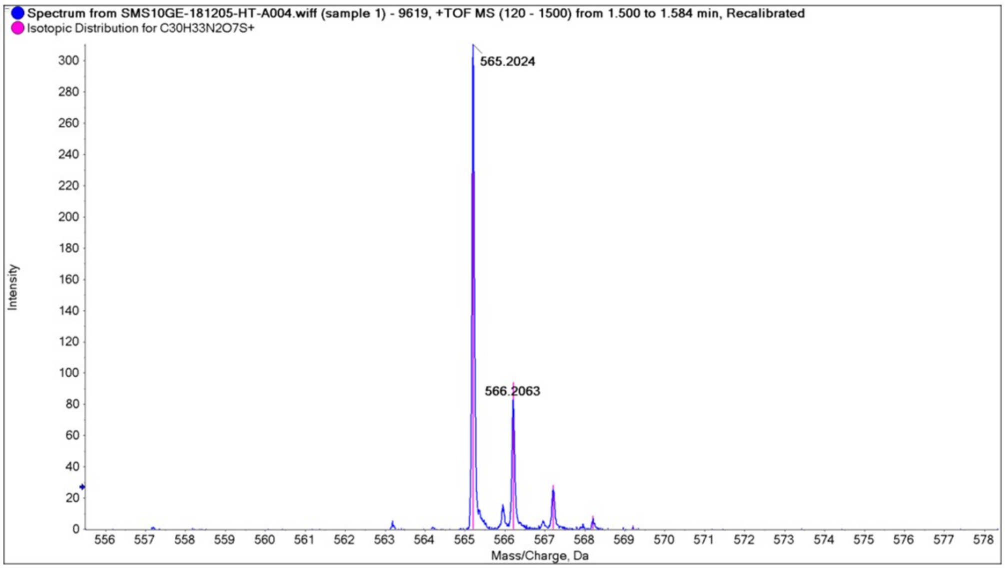

1/1

Figure S7. HRMS certificate of analysis of [HEL-SO_3H][BF_4]

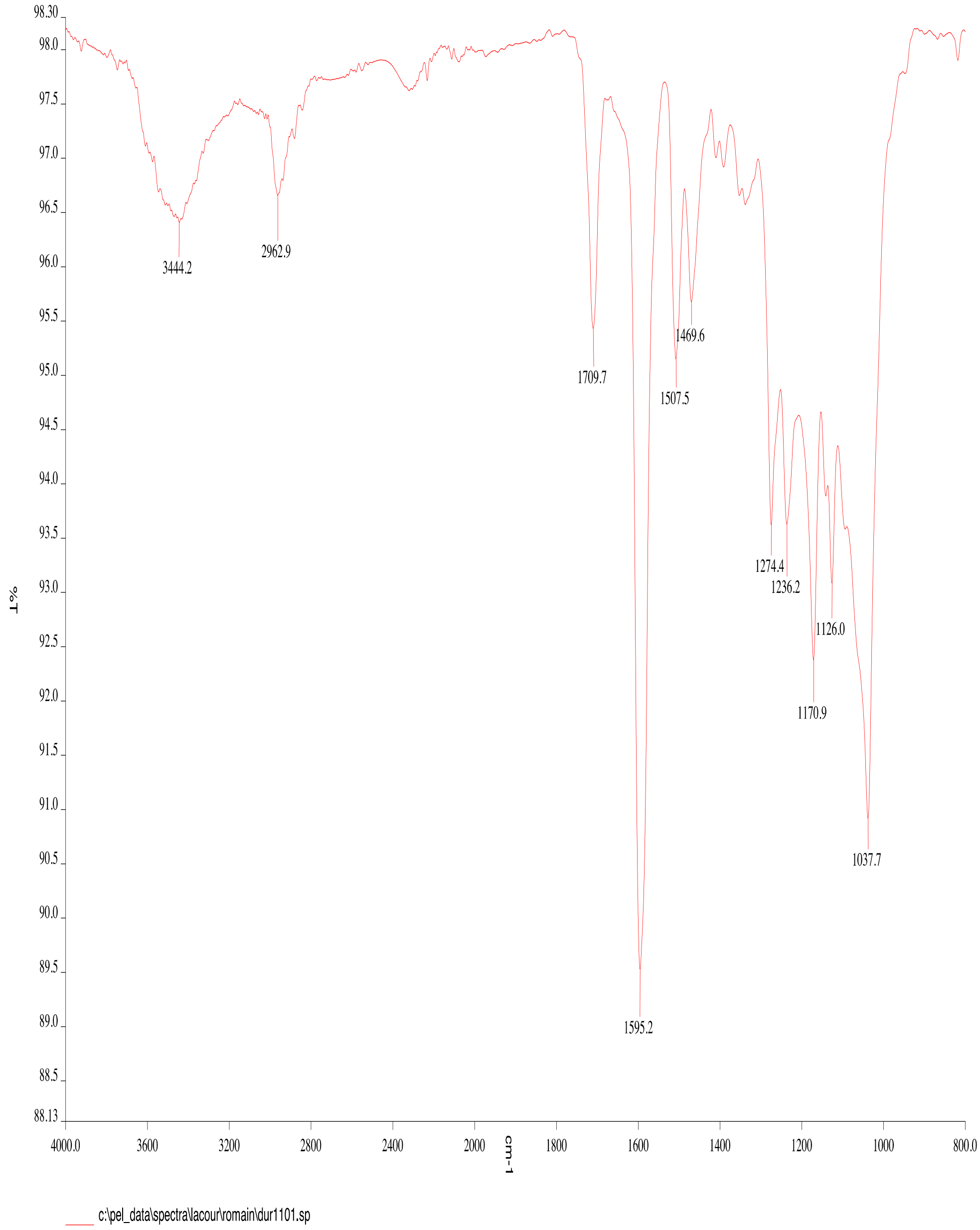

Figure S8. FT-IR analysis of [HEL-SO_3H][BF_4]

3. Water solubility study of $[HEL-AMH]^+$ and $[HEL-SO_3H]^+$

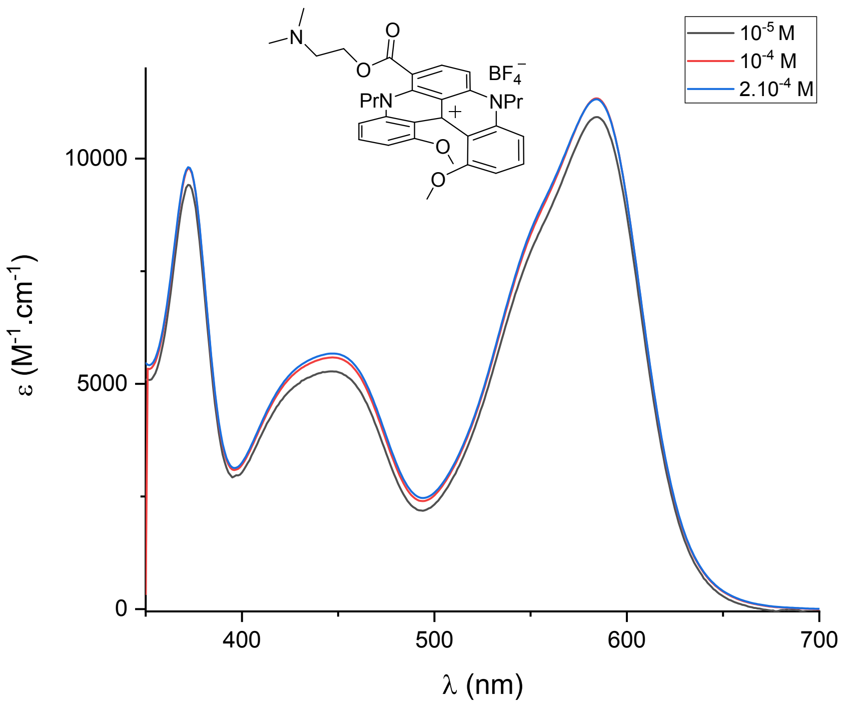

Figure S9. Water solubility control of [HEL-AMH][BF_4] in PBS

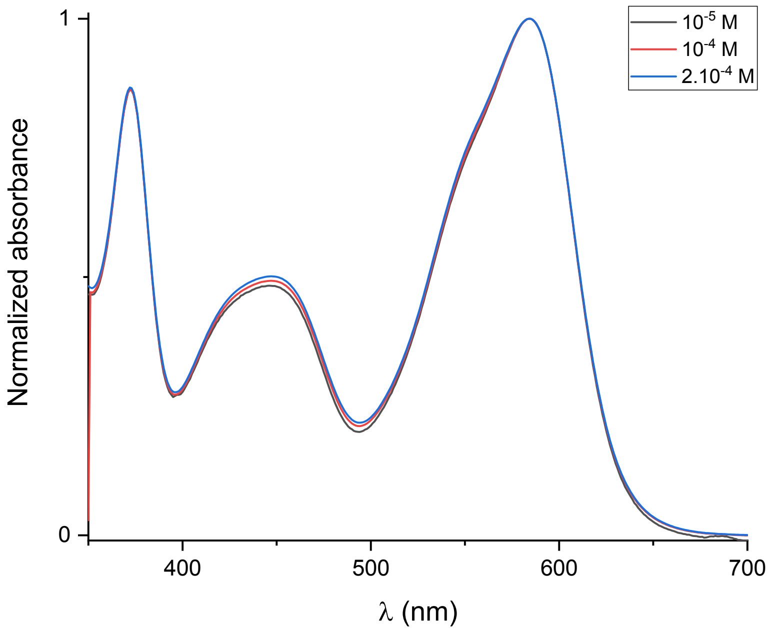

Figure S10. Aggregation control study of [HEL-AMH][BF_4] in PBS

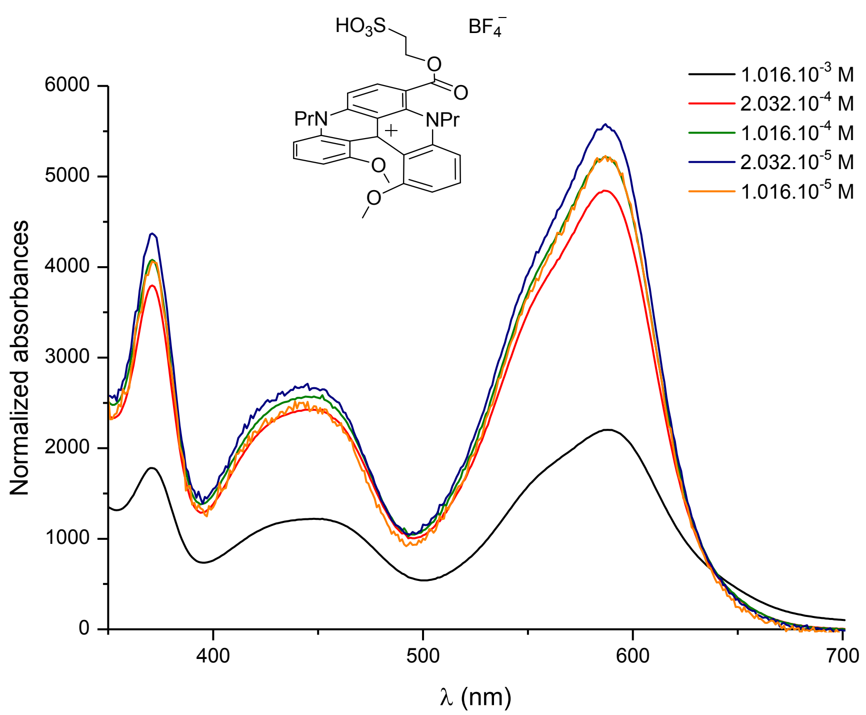

Figure S11. Water solubility control of [HEL-SO_3H][BF_4] in PBS

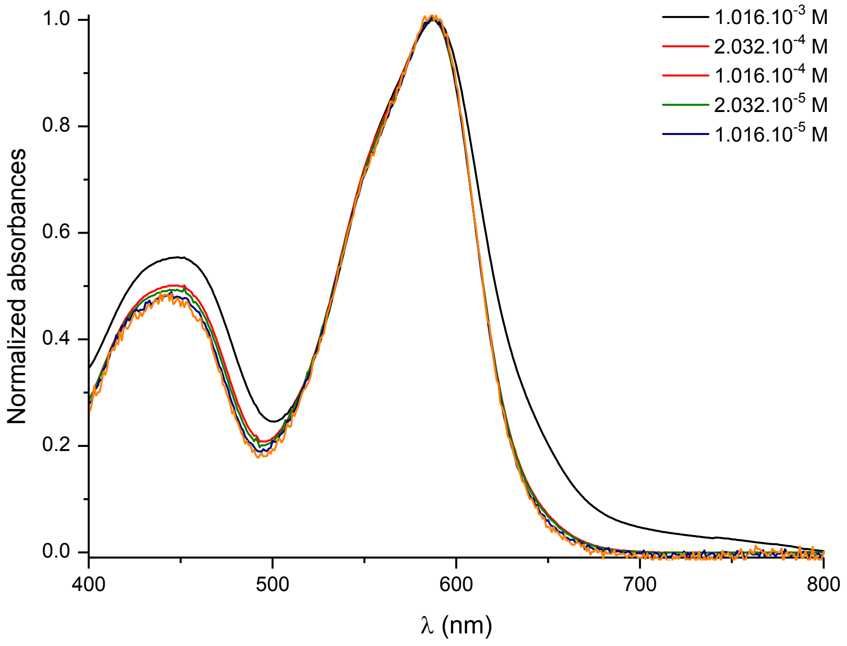

Figure S12. Aggregation control study of [HEL-SO_3H][BF_4] in PBS

4. Electrochemistry and ECL

Cyclic voltammetry was performed in PBS on a glassy carbon (GC) electrode using a µAutolab type III potentiostat. Before experiments, the electrode was polished with a 0.25 µm diamond paste (Struers). A platinum wire served as counter electrode and a Ag/AgCl/KCl 3M electrode was used as the reference electrode.

ECL emission was collected with a Hamamatsu R5070 photomultiplier tube. The PMT detector was held at -750 V with a Hamamatsu C9525 high-voltage power supply and placed at a defined distance of few millimeters from the working electrode. The output signal was amplified by a Keithley 6485 Picoammeter, then it was acquired *via* the second input channel of the µAutolab type III potentiostat. ECL spectra were recorded with a Princeton Instruments Acton SpectraPro 2300i after the CCD camera, cooled to -110°C with liquid N_2. The optical fiber connected to the device was located at a defined distance of few millimeters from the working electrode

5. Spectroscopic, electrochemical and ECL properties of the dyes

Table S1. Spectroscopic, electrochemical and ECL properties of the dyes

ECL dye	Photoluminescence		Electrochemistry and ECL		
	λ_{max} (nm)	Φ_{PL} (%)	E_{ox} (V) *vs.* Ag/AgCl/KCl 3M[e]	Φ_{ECL}^{TPrA} (%)	Φ_{ECL}^{Self} (%)[i]
$[HEL\text{-}AMH]^+$	**650**	21[b]	0.8, 1.33[f]	7.3[g]	-
$[Ru(bpy)_3]^{2+}$	**620**	3[c]	1.08	100	100
$[Ir(ppy)_2(pt\text{-}R)]^+$	476, **503**[a]	8.8[d]	1.14[f]	157[h]	10
$[Ir(df\text{-}ppy)_2(pt\text{-}R)]^+$	453, **483**, 515[a]	15.7[d]	1.5[f]	26[h]	12

[a] The iridium dyes were dissolved in DMF and diluted to 10 µM in a 0.1 M PBS solution (pH 7.4).[3] [b] Photoluminescence quantum yield in PBS relative to cresyl violet. [c] Photoluminescence quantum yield in 'ProCell' phosphate buffer used in commercial ECL instruments.[3] [d] Photoluminescence quantum yield in PBS. [e] Potentials measured in PBS on GC electrode. [f] Peak/plateau potential of the irreversible oxidation waves. [g] ECL efficiency measured by adding TPrA in PBS and relative to $[Ru(bpy)_3]^{2+}$. [h] ECL efficiency reported by Francis and workers (*Chem. Sci.*, 2019, 10, 8654). [i] Self-enhanced ECL efficiency relative to $[Ru(bpy)_3]^{2+}$, measured in PBS containing a constant concentration of 10^{-4} M $[HEL\text{-}AMH]^+$ and in presence of different iridium or ruthenium dyes at the same 10^{-4} M concentration on GC electrode (i.e. without adding TPrA).

t

6. Mechanism of oxidation/dissociation of the tertiary amine moiety

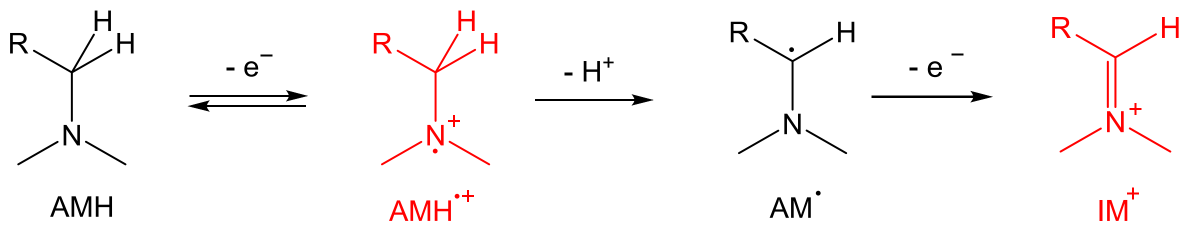

Scheme S1. Mechanism of formation of the different radicals after electron-transfer reactions and deprotonation of the tertiary amine moiety.[4-8]

Abbreviations:

- [HEL-AMH]$^{+}$ (red emitter/coreactant): [4]diaza helicene cation with dimethylamine moiety
- HEL: diaza core of the [4]diaza helicene luminophore
- AMH: dimethylamino moiety of the [4]diaza helicene luminophore
- HEL$^{\bullet}$: oxidized diaza core of the [4]diaza helicene luminophore
- AMH$^{\bullet+}$: oxidized form of the dimethylamino moiety of the helicene luminophore
- AM$^{\bullet}$: neutral radical of the dimethylamino moiety of the helicene luminophore obtained after oxidation and deprotonation
- IM^{+}: iminium product obtained after electron-transfer reaction with the oxidized luminophore

7. Voltammetric characterization of the [4]diaza helicene [HEL-SO_3H]$^+$

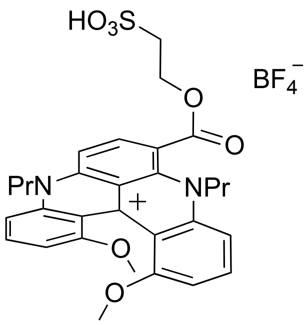

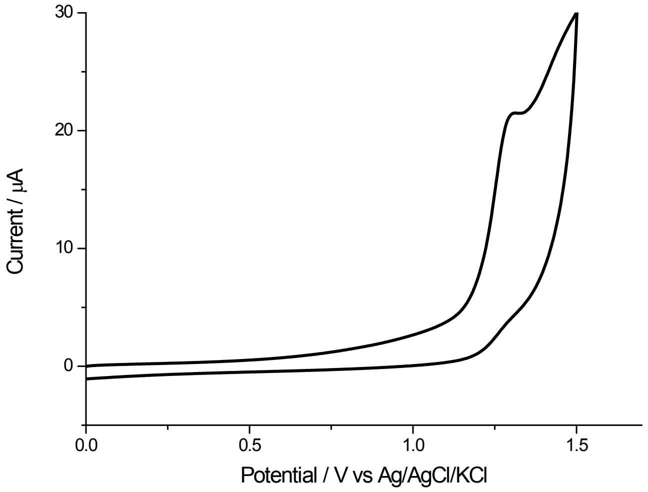

Figure S13. Cyclic voltammogram of a PBS solution containing 10^{-4} M [HEL-SO_3H]$^+$ on GC electrode. Scan rate: 0.1 V s^{-1}.

8. Comparison of the fluorescence and ECL spectra of [HEL-AMH]$^+$

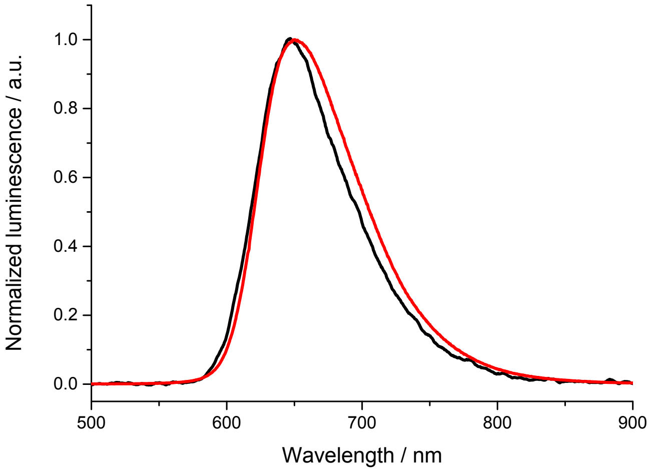

Figure S14. Normalized fluorescence (red curve) and ECL (black curve) spectra of [HEL-AMH]$^+$. ECL spectrum was recorded in a PBS solution containing a concentration of 10^{-4} M [HEL-AMH]$^+$. Applied potential: 1.35 V vs Ag/AgCl/KCl.

9. Voltammetric and ECL responses of $[HEL\text{-}AMH]^+$ in presence of $[Ir(df\text{-}ppy)_2(pt\text{-}R)]^+$

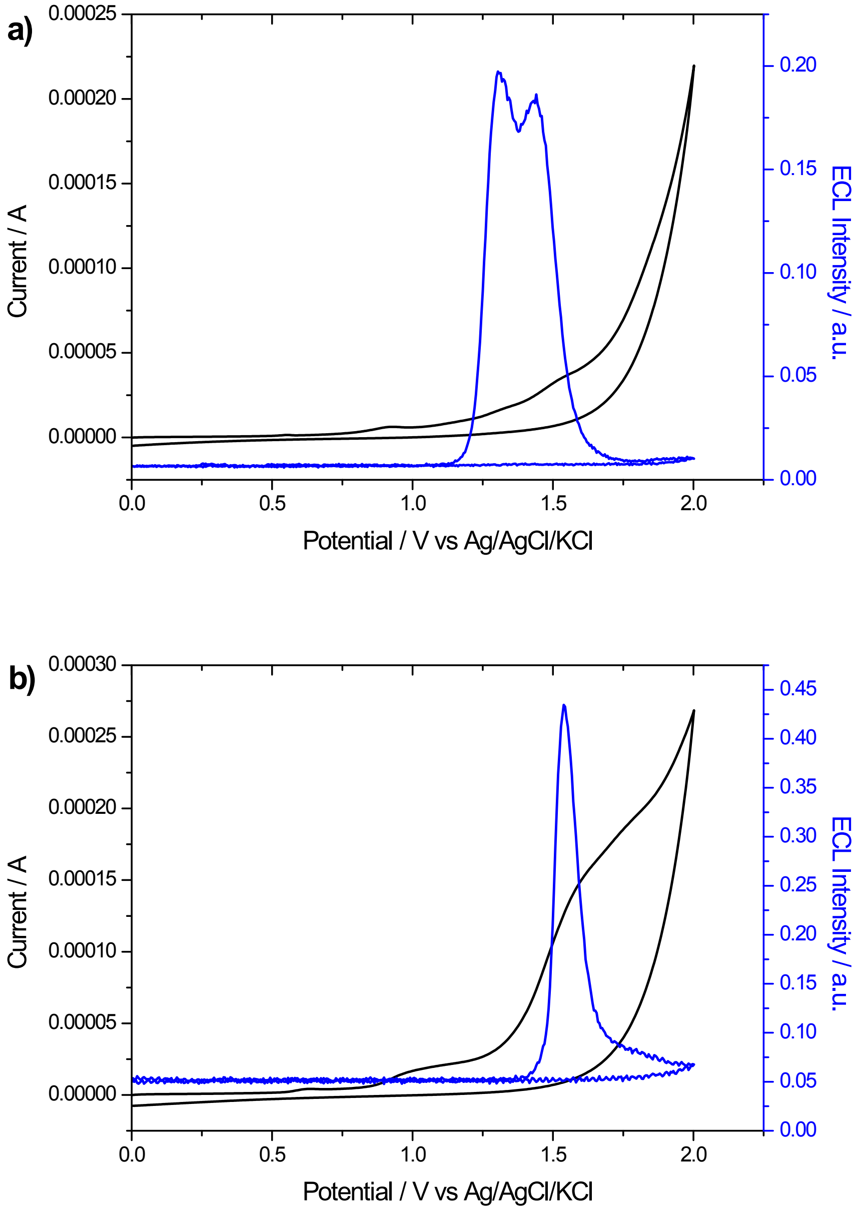

Figure S15. Voltammetric (black curve) and ECL signals (blue curve) of a PBS solution containing 10^{-4} M $[HEL\text{-}AMH]^+$ and a) 10^{-5} M or b) 10^{-3} M $[Ir(df\text{-}ppy)_2(pt\text{-}R)]^+$ on GC electrode. Scan rate: 0.1 V s^{-1}.

At the same scan rate, a similar irreversible oxidation as in Fig. S15a was observed in PBS (i.e. without $[HEL\text{-}AMH]^+$). Same behavior for $[Ir(ppy)_2(pt\text{-}R)]^+$.

10. ECL and fluorescence of $[HEL\text{-}AMH]^+$ in presence of $[Ru(bpy)_3]^{2+}$

a)

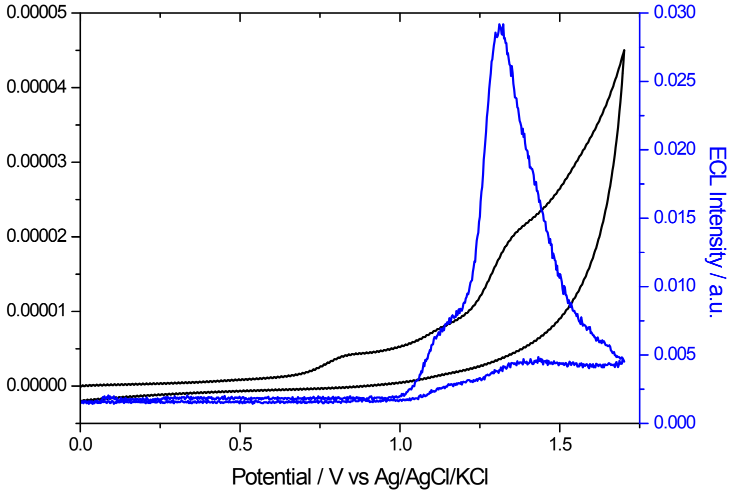

b)

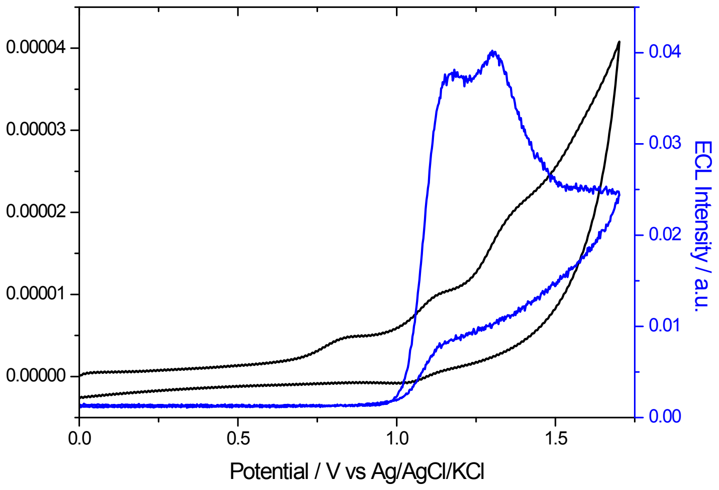

c)

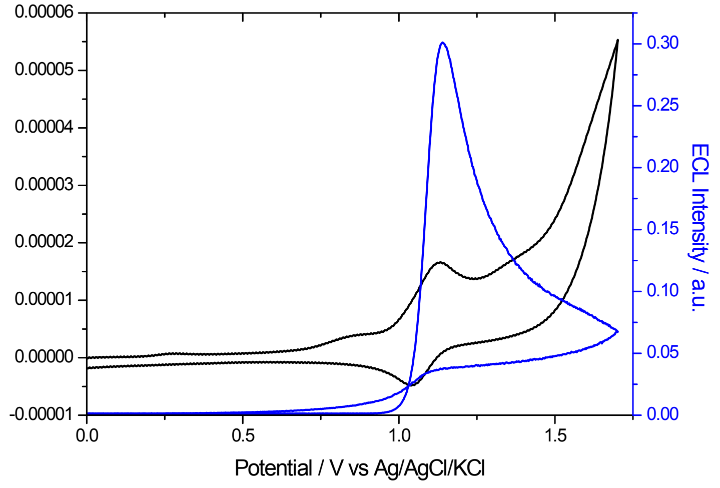

Figure S16. Voltammetric (black curve) and ECL signals (blue curve) of a PBS solution containing 10^{-4} M $[HEL\text{-}AMH]^{+}$ and increasing concentrations of $[Ru(bpy)_3]^{2+}$: a) 10^{-5} M, b) 10^{-4} M and c) 10^{-3} M. Scan rate: 0.1 V s^{-1}.

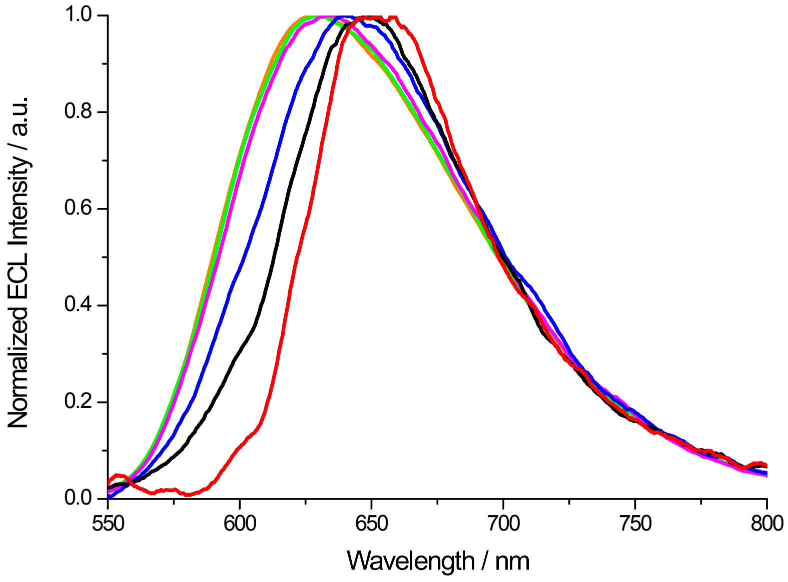

Figure S17. Normalized ECL spectra recorded in a PBS solution containing a constant concentration of 10^{-4} M $[HEL\text{-}AMH]^{+}$ and increasing concentrations of $[Ru(bpy)_3]^{2+}$: 0 (red curve), 1×10^{-5} M (black curve), 3×10^{-5} M (blue curve), 6×10^{-5} M (purple curve), 1×10^{-4} M (green curve) and 1×10^{-3} M (orange curve). Applied potential: 1.5 V vs Ag/AgCl/KCl.

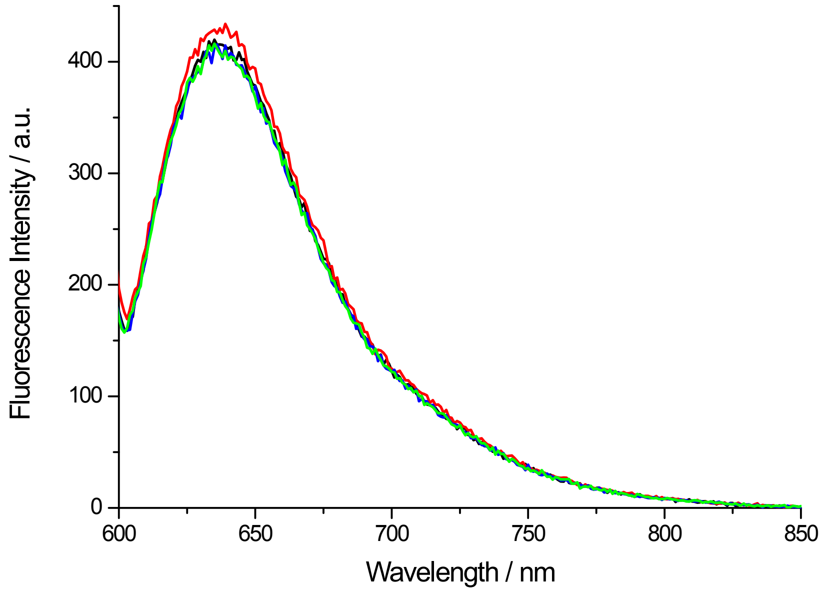

Figure S18. Photoluminescence emission spectra recorded in a PBS solution containing a constant concentration of 10^{-6} M $[HEL\text{-}AMH]^{+}$ and increasing concentrations of $[Ru(bpy)_3]^{2+}$: 0 (black curve), 10^{-7} M (red curve), 10^{-6} M (blue curve) and 10^{-5} M (green curve). λ_{exc} = 585 nm.

11. Voltammetric and ECL responses of $[HEL\text{-}AMH]^{+}$ in presence of $[Ir(ppy)_2(pt\text{-}R)]^{+}$

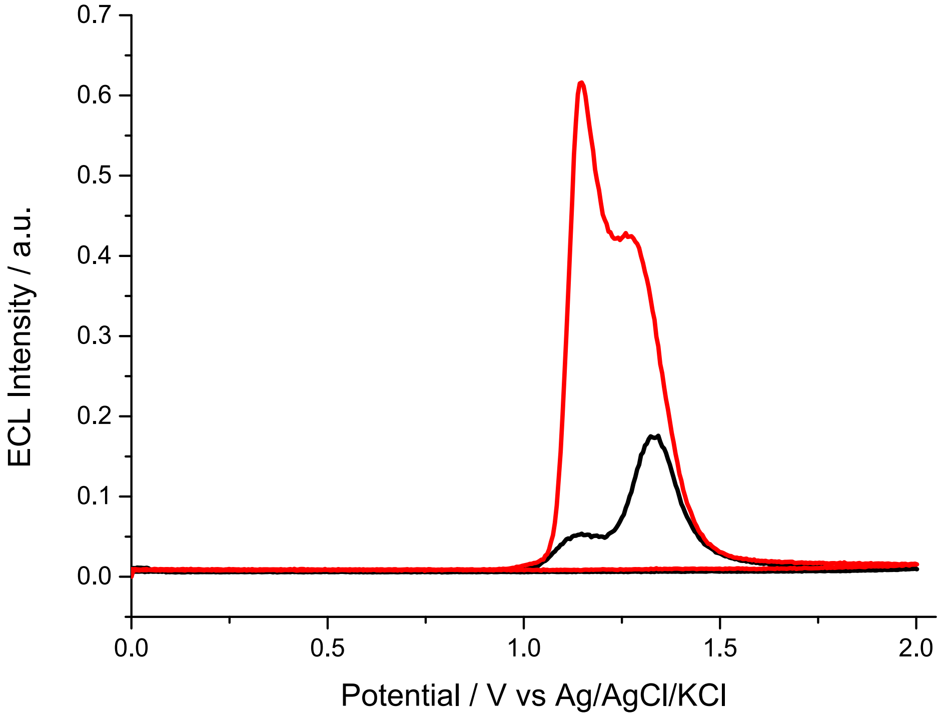

Figure S19. ECL signals recorded during voltammetric experiments in PBS containing 10^{-4} M $[HEL\text{-}AMH]^{+}$, and either 10^{-4} M (black curve) or 10^{-3} M $[Ir(ppy)_2(pt\text{-}R)]^{+}$ (red curve). Scan rate: 0.1 V s^{-1}.

10. References

1 S. Pascal, C. Besnard, F. Zinna, L. Di Bari, B. Le Guennic, D. Jacquemin and J. Lacour, *Org. Biomol. Chem.*, 2016, **14**, 4590-4594.

2 I. Hernández Delgado, S. Pascal, A. Wallabregue, R. Duwald, C. Besnard, L. Guénée, C. Nançoz, E. Vauthey, R. C. Tovar, J. L. Lunkley, G. Muller and J. Lacour, *Chem. Sci.*, 2016, **7**, 4685 - 4693.

3 L. Chen, D. J. Hayne, E. H. Doeven, J. Agugiaro, D. J. D. Wilson, L. C. Henderson, T. U. Connell, Y. H. Nai, R. Alexander, S. Carrara, C. F. Hogan, P. S. Donnelly and P. S. Francis, *Chem. Sci.*, 2019, **10**, 8654-8667.

4 R. M. Wightman, S. P. Forry, R. Maus, D. Badocco and P. Pastore, *J. Phys. Chem. B*, 2004, **108**, 19119-19125.

5 P. Pastore, D. Badocco and F. Zanon, *Electrochim. Acta*, 2006, **51**, 5394-5401.

6 D. Badocco, F. Zanon and P. Pastore, *Electrochim. Acta*, 2006, **51**, 6442-6450.

7 W. Miao, J.-P. Choi and A. J. Bard, *J. Am. Chem. Soc.*, 2002, **124**, 14478-14485.

8 R. Qiu, X. Zhang, H. Luo and Y. Shao, *Chem. Sci.*, 2016, **7**, 6684-6688.
